# Small Mediterranean coastal Lagoons Under Threat: Hydro-ecological Disturbances and Local Anthropogenic Pressures (Size Matters)

**DOI:** 10.1007/s12237-023-01182-1

**Published:** 2023-02-27

**Authors:** Viviana Ligorini, Eléa Crayol, Frédéric Huneau, Emilie Garel, Nathalie Malet, Marie Garrido, Louise Simon, Philippe Cecchi, Vanina Pasqualini

**Affiliations:** 1 Université de Corse Pascal Paoli, Campus Grimaldi, Corte, BP52, 20250 France; 2 CNRS, UAR 3514 Stella Mare, Cordon Lagunaire de la Marana, lieu-dit U Casone, Biguglia, 20620 France; 3 CNRS, UMR 6134 SPE, BP 52, Corte, 20250 France; 4 Ifremer, Laboratoire Environnement Ressources Provence-Azur-Corse (LER/PAC), Implantation de Bastia, Z.I. Furiani, Immeuble Agostini, Bastia, 20600 France; 5 Environmental Agency of Corsica, 14 Avenue Jean Nicoli, Corte, 20250 France; 6 MARBEC, Univ. Montpellier, CNRS, Ifremer, IRD, Montpellier, France

**Keywords:** Small Mediterranean coastal lagoons, Threats, Management, Ecological functioning, Hydrogeology, Phytoplankton

## Abstract

Mediterranean coastal lagoons are affected by multiple threats (demographic pressures, eutrophication, climate change) expected to increase in the future and impact the ecosystem services provided. Conservation norms and scientific studies usually focus on large lagoons (> 0.5 km^2^) due to their economic importance, while they ignore smaller lagoons. These are poorly understood and often unprotected, despite their prevalence within the Mediterranean region and their importance. Qualitative and quantitative characterisation of small lagoons, in terms of functioning and sensitivity to global and local changes, are needed to develop appropriate management strategies. For this purpose, this work provides the first inventory of all Corsican lagoons and has investigated three of them of small size (Arasu, Santa Giulia, Balistra), characterised by contrasting anthropogenic contexts (highly modified/disturbed, medium disturbance, *quasi*-pristine). At the regional level, 91 of the 95 lagoons identified are < 0.5 km^2^, making Corsica a good example for the study of small Mediterranean lagoons. The three case studies showed differences in their seasonal biogeochemical cycles and phytoplankton communities (biomass, diversity, photosynthetic efficiency). Arasu and Santa Giulia lagoons showed an increase in watershed urbanisation (+ 12% and + 6% in 30 years), high phytoplankton biomass, low diversity and blooms of potentially harmful dinoflagellates. Conversely, Balistra lagoon showed a good status overall, but some anthropogenic pollution sources within its watershed. This study demonstrates the importance of small lagoons at regional and Mediterranean scale, and provides knowledge on studied local sites but also potential applications elsewhere. The importance of an integrated approach considering lagoons within their adjacent connected systems (watershed and sea) and anthropogenic contexts is highlighted.

**Supplementary Information:**

The online version contains supplementary material available at 10.1007/s12237-023-01182-1.

## Introduction

Coastal lagoons are water bodies, generally parallel to the coastline, separated from the sea by a barrier but connected at least intermittently to the marine environment through one or more restricted inlets (Kjerfve 1994). They are very productive ecosystems and biodiversity hotspots, and provide numerous ecosystem services (Loureiro et al. 2006; Niedda and Greppi 2007; Barbier et al. 2011). Coastal lagoons are subjected to increasing anthropogenic pressures, i.e. urbanisation of the coastal zone that induces increase in water use and habitat loss (MWO 2018), nutrient inputs from the watershed that lead to eutrophication (Cloern 2001; Loureiro et al. 2006), and the effects of climate change, notably in the Mediterranean region (MedECC 2020; Cos et al. 2022). The development of effective management strategies for coastal lagoons is necessary, as they are increasingly subjected to threats which may result in their degradation (Pérez-Ruzafa et al. 2011; Ferrarin et al. 2014) and are experiencing continuous loss worldwide (Birch et al. 2022 and references therein).

Phytoplankton is considered a relevant indicator of ecological status and ecosystem functioning and it is often a central element in management strategies and norms (E.C., 2000; Birk et al. 2012). In coastal waters phytoplankton responds rapidly to any environmental changes, and alterations in its structure or dynamics have cascade effects on higher trophic levels (Cloern 1999; Loureiro et al. 2006; Armi et al. 2010; Goberville et al. 2010). Recently, shifts from diatoms to potentially harmful dinoflagellates have been observed in multiple environments and linked to variations in salinity, temperature and nutrient loads (Aligizaki et al. 2009; Collos et al. 2009; Xiao et al. 2018; Trombetta et al. 2019; Fischer et al. 2020). Moreover, changes in timing and increased frequency and magnitude of proliferations of harmful species, known as Harmful Algal Blooms (HABs), have been observed worldwide in recent decades, and especially in the Mediterranean region (Hallegraeff 2010; Kudela et al. 2015; Trombetta et al. 2019; Ligorini et al. 2022a).

To fully understand the functioning of coastal lagoons, it is necessary to take into account their hydro-climatic context and their connectivity to surrounding water bodies i.e. exchanges with the sea (Lloret et al. 2008; Biggs et al. 2015; Jones et al. 2018; De Wit et al. 2020) and continental inputs from surface waters and groundwater (Erostate et al. 2018, 2019, 2020; Jaunat et al. 2019). These elements are important to understand past and current pressures, such as watershed urbanisation, through the assessment of nutrient inputs (Erostate et al. 2018), and to be able to plan management strategies and corrective measures. Elevated nitrate (NO_3_^−^) concentrations in waters are unequivocal markers of anthropogenic pressures, mostly deriving from synthetic fertilizers and from septic tanks (Re et al. 2017; Vystavna et al. 2017; Erostate et al. 2018). Local hydrological connectivity is deeply influenced by both climatic constraints and human activities through the withdrawal of the initial water resource for any purposes, and the restitution of more-or-less treated water to the hydrographic network. Human interventions (e.g. opening or closure of outlets), local conditions (e.g. winds, currents) and global pressures (sea level rise) may also control the intensity of exchanges with the marine environment.

The main sources of knowledge on European coastal lagoons are the regular scientific surveys carried out in the context of the Water Framework Directive (WFD, European Union (EU) 2000; Loureiro et al. 2006; Renzi et al. 2019). The WFD does not provide a legal definition of coastal lagoons, and national interpretations of the directive often add a surface-size limit set at 0.5 km^2^ for its application to transitional waters (e.g. ISPRA 2014 for Italy; SDAGE 2021 for France - *Bassin de Corse*). Since smaller coastal lagoons are not included in the regulation, they remain neglected, poorly understood and ultimately poorly or even not at all managed. Among Mediterranean coastal countries, at least four (Greece, Italy, Spain, Turkey) have lagoons smaller than 0.5 km^2^ (Cataudella et al. 2015), but quite apart from the WFD and its size-limit, scientific studies usually focus on large lagoons, since they historically sustain economic activities. There have been a few studies on these small systems, but they are mostly very local case studies (Tuncel et al. 2007; Camacho et al. 2012; Mitchell et al. 2017; Doughty et al. 2019). However, small lagoons are extremely valuable and common in various parts of the world, like in the western coasts of Africa and North America, in South eastern American and Australian coasts, and especially in the Mediterranean region (Suzuki et al. 1998; Dye and Barros 2005; Tuncel et al. 2007; Moreno et al. 2010; Camacho et al. 2012; Mitchell et al. 2017; Doughty et al. 2019). They may also be particularly vulnerable to increasing pressures such as human activities and climate change, in relation with their small size, and hence have less resilience in the face of disturbance (Doughty et al. 2019). The ratio of perimeter to area increases as the size decreases, increasing edge effects and strengthening the coupling between the lagoon and its watershed’s land use and hydrology. A better understanding of the structure and function of these highly reactive systems can inform more effective management to mitigate the negative consequences of climate and land use change. Currently, there is no extensive evidence of differences between small and large lagoons, due to lack of knowledge and hence difficulties in comparison. However, it has already been demonstrated that, for continental waters, small lakes host higher biodiversity compared to larger lakes and play an important (and neglected) role in large-scale processes; they are also more active and dynamic than large ones, potentially increasing the threat posed to them by climate change (Downing 2010). Given all these premises, it is clear that small lagoons deserve to be considered a priority interest with regard to their conservation. In Europe, apart from the WFD, another norm defines conservation objectives for coastal lagoons, with a different approach: the Habitat Directive (HD, European Union (EU) 1992). This directive does not include a size limit, so the question of small lagoons arises: most of them are not only without any protection status, but they are not even acknowledged, so inventories, quantification and qualification of these systems is needed (Latron et al. 2022).

An excellent example with regard to this major challenging goal of the conservation of coastal lagoons, especially in the Mediterranean region, is Corsica Island (France), located in the north-western Mediterranean Sea. The island has more than 200 coastal wetlands, mostly brackish coastal lagoons, highly diverse and is a good example for the study of small-sized coastal lagoon environments (Ghilardi 2021; Di Rita et al. 2022). Only the four largest lagoons are well known, since they fit with the WFD criteria and have been the subject of numerous scientific studies and monitoring surveys. In the legislative and social-ecological context previously described, managers are drawing attention to the need to focus on underrated small lagoons. Specifically, research is needed to (1) improve qualitative and quantitative characterisations of small lagoons, including their hydrology and ecology, (2) predict how climate change will affect small lagoons and (3) inform management and restoration decisions. In this study, we have sought to answer some of these questions, firstly, by a description of small coastal lagoons at regional level, since their importance is still underestimated, and secondly, by the investigation of three case studies in Corsica Island. The three small lagoons chosen, Arasu, Santa Giulia and Balistra, are of comparable size (e.g. 0.25 km^2^) but present three different anthropogenic contexts and histories and contrasting current human pressures. We have considered their connection to adjacent systems, in order to better characterise the ecosystems in their hydrogeological and anthropogenic contexts. To do so, anthropogenic tracers from the watersheds of the three case studies were detected to describe sources of human pressure. Then, seasonal samplings over two consecutive years were performed in the lagoons to analyse environmental characteristics and phytoplankton variations, to assess seasonal patterns and to detect relationships between abiotic and biotic factors.

The main aim of the study was to raise awareness of these little known small-surface systems. To do that, firstly, we contextualised the importance of small-sized lagoons at regional and Mediterranean scales. Secondly, for the three case studies two main questions are addressed: (i) how do these three small lagoons, of comparable size but with different levels of anthropogenic impact in their watersheds, differ in terms of their physical and ecological functioning? (ii) what implications do these differences have for the lagoons’ vulnerability towards climate change and other environmental stressors? Ultimately, our aim is to provide new knowledge to stimulate the development of management strategies dedicated to small-sized coastal lagoons at regional and global scale.

## Materials and Methods

### Lagoons at Mediterranean and Regional Scale

A qualitative description of the large-scale distribution of lagoons in the Mediterranean basin was performed on the basis of available data (Cataudella et al. 2015) and used for comparison with coastal lagoons around the Corsican coast. We compiled data from the Environmental Agency of Corsica (OEC; unpublished data) and the *Pôle Relais Lagunes Méditerranéennes – Tour du Valat* (PRLM/TDV; unpublished data) on the number, surface areas, perimeter, ownership and management of all Corsican lagoons. The water masses considered in this study are classed as “Mediterranean coastal lagoons,” according to the definition of the habitat 1150*-2, provided by the European Directive 92/43/CEE (Habitats Directive) applied in France (Barre et al. 2020). Mapping of coastal lagoons (PRLM/TDV; unpublished data) were obtained through the analysis of satellite images from Sentinel-2A and Sentinel-2B (20-m resolution) systems (https://glovis.usgs.gov/), over a selected hydrological period (2018–2020) using the ArcGIS 10.3 software.

### Three Case Studies

#### Study Sites

**Fig. 1:**
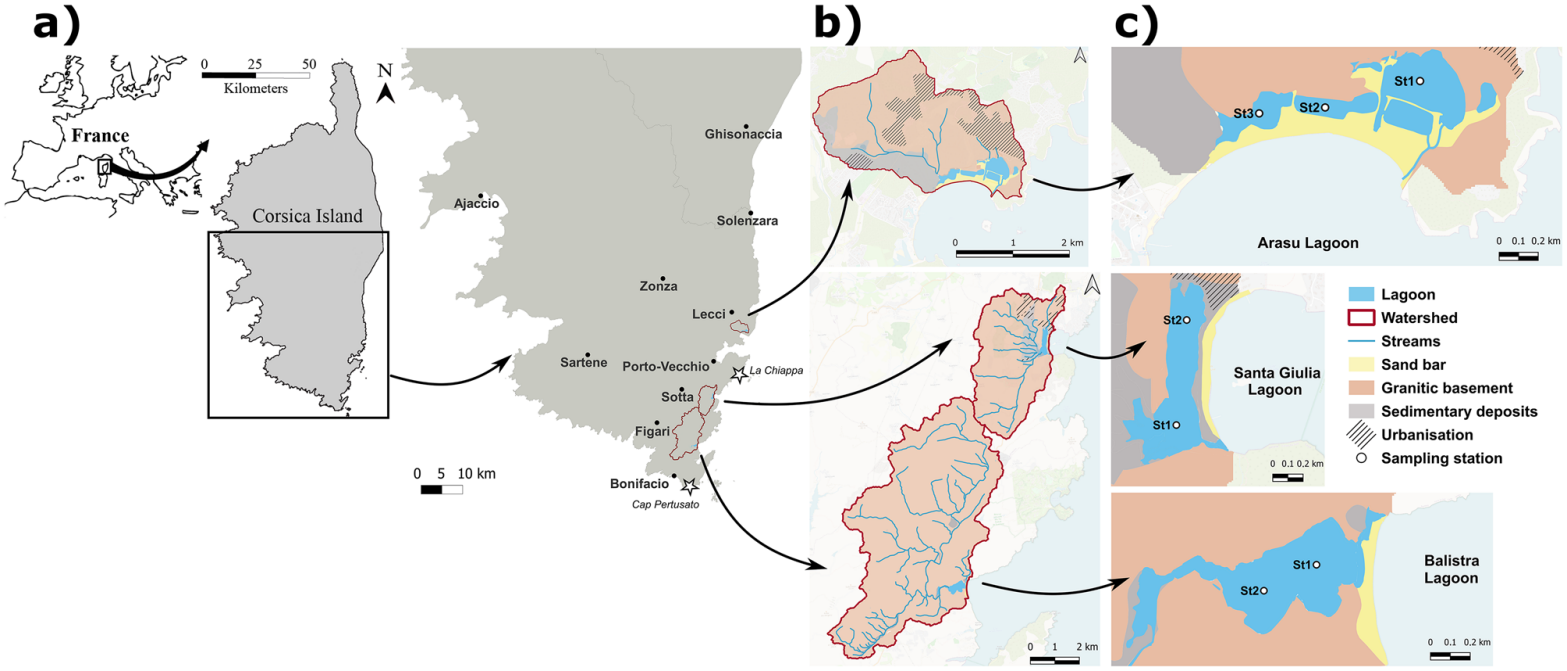
**a** Study sites location. Stars on the map correspond to the locations of meteorological stations. **b** Main geological elements and urbanisation of the lagoons’ watersheds. **c** Sampling stations within each coastal lagoon

The three lagoons in the study are located in the south-eastern part of the island (Fig. 1a). The contours of the three watersheds were drawn using a digital elevation model (resolution of 5 m) and georeferenced on QGIS software (version 3.22.5 “Białowieża”). Geological maps (1:50000) were retrieved from the *Bureau de recherches géologiques et minières* (BRGM) website (BRGM - InfoTerre), and simplified by grouping similar geological formations, resulting in three main geological descriptors: sand bar, granitic basement and sedimentary deposits (Fig. 1b, c).

Arasu lagoon (41°38′34″ N, 09°21′55″ E, Fig. 1b, c) covers a 0.26 km^2^ surface area and receives freshwater inputs from a 5 km^2^ watershed (OEC data), which is predominantly constituted of densely fractured and intensively weathered granite (72%). In the southern-lower part of the Arasu lagoon watershed, the geology is characterised by clay-sand deposits, favourable to the presence of a restricted alluvial aquifer, formed by Quaternary sediment deposits and hydraulically connected to the lagoon in this area. The main freshwater sources are three streams in the western part of the basin, while exchanges with the sea take place in the eastern part only, through a small channel, with a bifurcation (Fig. 1c). The lagoon has been considerably altered in the past, as during the 1960s a project for the creation of a marina was initiated. Once abandoned, the project still left certain major alterations, such as the presence of roads on dams crossing the lagoon and dividing its surface into three separate basins (Fig. 1c), communicating through small culverts underneath the causeways. Since sediment from the lagoon was dug out, the basins are of different depths, with the middle basin being the deepest (max. depth = 4 m) and a mean depth over the entire basin of 1.5 m. At present, the lagoon is owned by several owners, private and public, including the *Conservatoire du Littoral* (CdL). It has been designated under the Special Areas of Conservation (SAC) protection status as a Natura 2000 (N2000) site (Habitats Directive) and it is under the management of the *Collectivité de Corse* (CdC). Major threats for this basin are the growing urbanisation of the north-eastern side and tourism during the summer period (e.g. beach restaurant).

The shallow Santa Giulia lagoon (41°31′32″ N, 09°16′12″ E; max. depth = 1.5 m, mean depth = 0.3 m; Fig. 1b, c) extends for 0.23 km^2^ on a north-south axis (OEC data). The geology in the area of Santa Giulia lagoon is very densely fractured (79% granite), with many major clear regional lineaments. The lower watershed is covered by a thick sedimentary layer made of clayey sands that favours the infiltration of water and the rapid saturation of soils as well as the emergence of visibly flowing groundwater springs. The 15.5 km^2^ watershed provides freshwater inputs mainly at the southern end of the basin, near to the outlet of the lagoon, where small temporary creeks flow and are associated with a restricted alluvial aquifer, pumped through several wells and boreholes by local residents for agriculture and gardening purposes. The sea channel is also located in the southern part (Fig. 1b, c, OEC data). The northern part of the basin does not present a direct exchange with the sea nor strong freshwater inputs (Fig. 1c). The lagoon is owned by the CdL and managed by the Environmental Agency of Corsica (OEC) under its protection status (SAC as N2000 site, Habitats Directive). Major threats for this lagoon are the tourism activity around its borders and management difficulties as unauthorised interventions by users of the beach often occur to open the sea channel when obstructed by the accumulation of sand and litter. Small-scale artisanal fishing is carried out by a single fisherman and there is cattle grazing in the south-western area of the watershed (personal observation).

The Balistra lagoon (41°26′25″ N, 09°13′11″ E, Fig. 1b, c) covers 0.27 km^2^ and receives freshwater inputs from the Francolu river in the western part, from a total watershed of 37 km^2^ (OEC data). The Balistra lagoon watershed is mostly granitic (93%) and very densely fractured and weathered, favouring the creation, in the immediate vicinity of the mouth of the Francolu river, of a restricted alluvial aquifer made of coarse sand. This configuration shows a similar hydrogeological structure to that of Santa Giulia, with the emergence of multiple isolated groundwater springs and high porosity, favouring continuous groundwater flow contribution to the rivers and thus to the downstream lagoons.

The depth of Balistra lagoon reaches a maximum of 4.5 m and a mean over the basin of 2 m (Pergent-Martini et al. 1997). The sea channel is located in the north-eastern part and has a completely natural mode of functioning (Fig. 1). The lagoon is entirely privately owned and is not subject to any management measures at present despite its SAC protection status as a N2000 site (Habitats Directive). The lagoon is located in a barely urbanised area. Nevertheless, some threats do exist, such as a heavy influx of tourists during summer.

On the basis of the little information available, often from non-validated/unpublished scientific reports or non-scientific documents, the three lagoons present a decreasing gradient of current human pressures: Arasu is the most disturbed, Santa Giulia shows a medium status, mostly linked to usages and frequentation, and Balistra could be considered representative of a *quasi*-pristine condition.

#### Data Collection

To describe the meteorological context both long-term and over the sampling period, daily rainfall and mean air temperature were collected from the Météo-France database. La Chiappa (41°35′41″ N, 9°21′47″ E) and Cap Pertusato (41°22′29″ N, 9°10′42″ E) weather stations were chosen (for Arasu and Santa Giulia lagoons and for Balistra lagoon respectively), based on their proximity to the study sites (Fig. 1a). Cumulative monthly rainfall and mean monthly air temperature were calculated to characterise the meteorological context over the period 2019–2021 and for comparison with the standard reference period means established for France (1991–2020) according to the World Meteorological Organisation guidelines (WMO-No. 1203^©^ World Meteorological Organization 2017).

In order to characterise the progression of urbanisation and touristic pressure over the three watersheds, data on residential homes were collected from the *Institut National de la Statistique et des Études Économiques* website (^©^Insee, https://www.insee.fr) for various municipalities, depending on the lagoon considered. The municipalities taken into account are the following: Zonza and Lecci for the surroundings of Arasu lagoon, Porto-Vecchio and Bonifacio for the surroundings of Santa Giulia and Balistra lagoons, respectively. Data on principal and secondary residential homes were available from 1968 to 2018, with a 7-year frequency until 2006 and every 2 years afterwards. The CORINE land-cover map of catchments for 2018 and available previous years (1990, 2000, 2006 and 2012) were then defined (CORINE Land Cover) using three simplified classes (natural or semi-natural, agricultural and urbanised), based on land cover types automatically defined by the CORINE land-cover program, and compared in order to analyse the urbanisation progression rate over the last 30 years.

Surface water and groundwater were sampled to get an overview of the entire hydrological functioning of the three watersheds, with a focus on the interactions between surface water, groundwater and lagoons. Groundwater was sampled (see Fig. 3) in five springs in the upper part of the watersheds (1 Arasu, 4 Balistra), two hand-dug 1 m-depth piezometers close to the lagoons (1 Arasu, 1 Balistra), five wells (around 5-m depth) in the lower part of the watersheds (1 Arasu and 4 Santa Giulia) and three boreholes (40–90-m depth) in the Arasu watershed. Sampling was carried out during autumn 2020 after a dry hydrological year, and NO_3_^−^ concentrations in water samples were determined by Ionic Chromatography (ICS 1100 chromatograph Thermo Dionex, Sunnyvale, CA, U.S.A) at the Hydrogeology Department of the University of Corsica (Corte, France).

Seasonal samplings of lagoons water basins were carried out over the two consecutive years 2020 and 2021 in February (winter), May (spring), August (summer) and November (autumn). Seasonal subsurface (−0.2 m) water samples were hand-collected in the water basins at three sampling stations for Arasu lagoon and at two sampling stations for Santa Giulia and Balistra, between 7:30 and 10:30 a.m. (Fig. 1c). Sampling stations’ location and number were chosen to best quantify the spatial variability of the different basins. Triplicate measurements of salinity, temperature, turbidity and dissolved oxygen concentration were performed in situ at each station in subsurface with an YSI ProDSS multiparameter water quality probe. 1 L water samples were pre-filtered on a 200-µm mesh sieve and collected in plastic bottles for further nutrient, phytoplankton pigment and cytometry analyses. Ammonium (NH_4_^+^) concentrations were determined through the fluorescence method (Holmes et al. 1999) and nitrite (NO_2_^-^), nitrate (NO_3_^−^), dissolved inorganic phosphorus (DIP) and silicates concentrations by the colorimetric method (Aminot and Kérouel 2007) after filtration on Whatman GF/F filters (47 mm, porosity 0.7 µm) and water samples storage at −20°C until analyses, at the Mediterranean Institute of Oceanography (MIO) research laboratory (Marseille, France). The use of different methods for the determination of inorganic nutrients concentrations was imposed by technical reasons and does not compromise comparability, precision and accuracy of the estimates (Burke et al. 1989; Aminot and Kérouel 2007; Alahi and Mukhopadhyay 2018; Leruste et al. 2019).

Phytoplankton communities were analysed based on their biomass, abundance, diversity and photosynthetic activity. Chlorophyll *a* (Chl *a*, µg L^−1^) was used as the proxy of phytoplankton biomass. To determine its concentration, water subsamples (250 mL) were filtered on Whatman GF/F filters (25 mm, porosity 0.7 μm) and further stored at −20°C until spectrofluorometric analyses (Neveux and Lantoine 1993). To identify microphytoplankton (> 20 µm) community composition and quantify abundances, 50 L of surface water were filtered at each station through an Apstein plankton net (20 µm mesh) and 100 mL of concentrated sample were fixed with formaldehyde at 2.5% final concentration. All samples were then examined according to the Utermöhl method (Utermöhl 1958; AFNOR 2006) with an inverted microscope (Olympus^®^ CKX41). At least 400 cells were counted (estimation error within ± 10% limits) (Lund et al. 1958; Uehlinger 1964). Identification was performed at lowest possible taxonomic level with verification according to several books (Sournia 1986; Throndsen 1997; Horner 2002; Avancini et al. 2006; Hoppenrath et al. 2009; Steidinger and Meave del Castillo 2018) and databases such as the World Register of Marine Species or AlgaeBase (http://www.marinespecies.org/, https://www.algaebase.org/, databases available online). In order to characterise community functional diversity, the smallest fraction of phytoplankton communities was also taken into account and analysed through flow cytometry. A total of 1.8 mL of water samples were fixed with glutaraldehyde (0.25% final concentration) and with addition of Pluronic (Poloxamer 188) at a final concentration of 0.01%, as described in Marie et al. (2014). Then, fixed samples were stocked at −80 °C and used to perform analyses according to the method detailed in Marie et al. (2014). Different groups could be detected based on their dimension and fluorescence: nanophytoplankton (NANO; > 2 µm) and, among the picophytoplankton (< 2 µm), autotrophic picoeukaryotes (PEUK), phycocyanin-rich picocyanobacteria (PC-cyan) and phycoerythrin-rich picocyanobacteria (PE-cyan) populations were identified.

Photosynthetic activity efficiency was assessed with a Pulse-Amplitude-Modulated fluorimeter (Phyto-PAM Plankton Analyser; Heinz Walz GmbH, Effeltrich, Germany) on samples dark-adapted for at least 30 minutes. Samples were kept in a cooler between 10 and 15 °C and analysed between 2 and 6 h from field collection in order to respect recommended storage conditions for Phyto-PAM fluorimetric analyses (Garrido et al. 2013a). The ratio Fv/Fm, maximum quantum yields of Photosystem II (PSII), was used to assess the health status of the phytoplankton community (Garrido et al. 2013a). It was determined by the equation: Fv/Fm = (Fm-F0)/Fm, where Fm is the maximum fluorescence emitted under a saturating pulse of light (4000 µmol photons m^−2^ s^−1^) and F0 is the intrinsic initial fluorescence under non-actinic light when all PSII centres of reaction are opened and potentially available for electron transport (Garrido et al. 2013a). Phyto-PAM analyses were performed in triplicate.

#### Statistical Analysis

Data analysis was performed using R statistical software version 4.0.2 (RStudio Team 2016). Mean seasonal values were calculated for each parameter measured in triplicate: salinity, temperature, turbidity and dissolved oxygen concentration measured in situ, and Fv/Fm measured at laboratory. Abundances by classes of microphytoplankton (Bacillariophyceae or Diatoms, Dinophyceae or Dinoflagellates, Cyanophyceae, Cryptophyceae, Chlorophyta and Others) or groups of pico- and nano-phytoplankton (PC-cyan, PE-cyan, PEUK and NANO) were considered by year and season. Microphytoplankton community was also described through the calculation of diversity indexes (species richness, Shannon’s Diversity Index), based on single taxonomic entities identified during counting and considered as taxonomic units. A list of all species or taxonomic units identified can be found in Table S1 (Supplementary).

All data were previously log_10_(x+1) transformed to normalise data, reduce effects of extreme values and meet ANOVA conditions.

Spatial and seasonal differences within lagoons were assessed through analysis of variance (ANOVA) tests for each variable of interest. When significant (*p* < 0.05), differences were further investigated through post-hoc Tukey’s test. For some variables, since normality was not achieved despite transformation, a Kruskal-Wallis (K-W) non-parametric test was applied and eventual significant differences were further tested through Dunn’s post-hoc test. Then, a global analysis was performed, with visualisation through Non-metric Multidimensional Scaling (NMDS) of abiotic parameters (water temperature (°C), salinity, turbidity (FNU), dissolved oxygen concentration (%), silicates (µM), DIP (µM), NO_3_^−^ (µM), NO_2_^−^ (µM) and NH_4_^+^ (µM)) and biotic variables (Chl *a* (µg L^−1^), Fv/Fm, Diatoms (cell L^−1^), Dinoflagellates (cell L^−1^), Cyanophyceae (cell L^−1^), Cryptophyceae (cell L^−1^), Chlorophyceae (cell L^−1^), Others (cell L^−1^), PE-cyan (cell L^−1^), PC-cyan (cell L^−1^), PEUK (cell L^−1^), NANO (cell L^−1^), species richness and Shannon’s Diversity Index) separately. To test potential effects of season, lagoon and stations, datasets were investigated through the permutational multivariate analysis of variance (PERMANOVA) test and significant differences (*p* < 0.05) were afterwards detailed through the pairwise post-hoc test.

Finally, relationships between biological and environmental variables were analysed through Spearman’s ranks order correlation for each lagoon.

## Results

### Small Lagoons at Mediterranean and Regional Scale

**Fig. 2:**
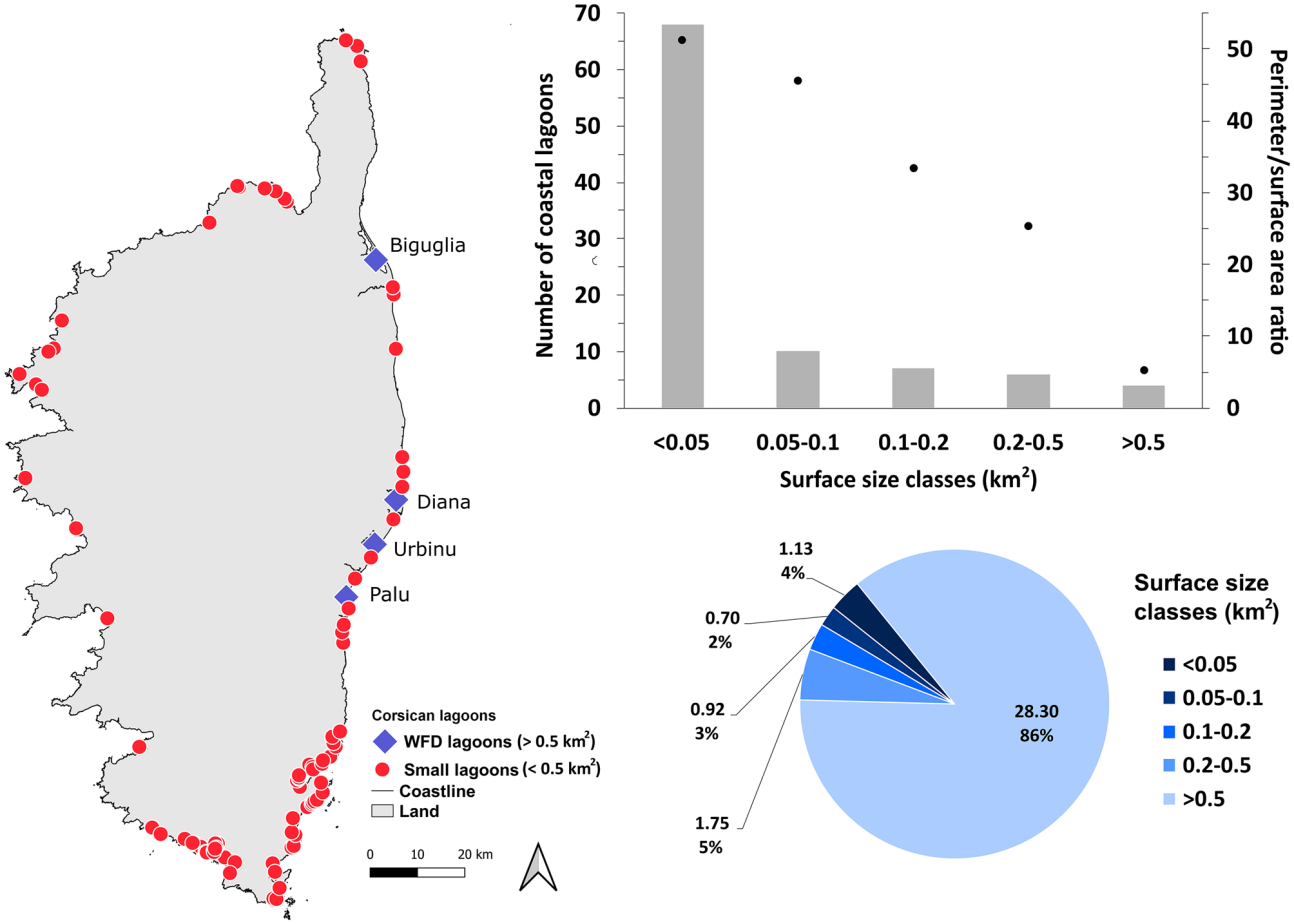
Coastal lagoons in Corsica: locations along the coastline are represented with size-classification according to the WFD limits (left); on the bar chart (top right), number (left scale, bars) and perimeter (km)/surface area (km^2^) ratios (right scale, points) according to size classes are shown; finally, on the pie chart (bottom right) a representation of surface proportions for each size class is reported (total surface (km^2^) and percentage for each size class are shown)

The Mediterranean coastline measures 46,000 km and hosts approximately 400 lagoons, ranging from 0.02 to 780 km^2^ (Cataudella et al. 2015). No clear estimation of coastal wetlands has been established for the Mediterranean region; however, the estimated total lagoon surface area is around 6,470 km^2^ according to literature (Pearce and Crivelli 1994; Cataudella et al. 2015). Apart from knowledge emerging from literature, results from recent studies of the PRLM/TDV, including small-surface lagoons, determined that 95 lagoons can be found in Corsica alone, for a total surface area of 32.81 km^2^ (Fig. 2). So, Corsica has 95 lagoons spread over its 1,047-km of total coastline, or an average of one lagoon per 11 km, compared to one lagoon per 115 km of coastline for the Mediterranean in general. In Corsica, only 4 lagoons have surface areas greater than 0.5 km^2^, allowing their integration in the WFD, and they cover altogether 28.30 km^2^ (Fig. 2). The other 91 lagoons, smaller than 0.5 km^2^, represent around 14% (4.51 km^2^) of the total lagoon surface area on the island, and among them, 68 cover less than 0.05 km^2^ (Fig. 2). Due to their small surface area, small lagoons show a high perimeter/surface area ratio, reaching 51.30 for the < 0.05 km^2^ size class, against 5.23 for the four largest lagoons (Fig. 2). The total perimeter of small-sized lagoons is even longer than that of the four WFD lagoons, measuring 165.46 vs 148.10 km, respectively.

### Three Case Studies

#### Hydro-climatic Context and Watershed

Overall, analysis on meteorological conditions highlighted a similar pattern for both meteorological stations over the standard reference period 1991–2020, with a 3-month dry summer season from June to August (Supplementary Fig. S1a). Compared to this period, the overall 2019–2021 period was drier: La Chiappa station in particular was drier than Cap Pertusato station, showing a 5-month dry summer season from May to September, and two winter dry months, February (21 mm mean cumulative rainfall; Supplementary Fig. S1b) and March (22 mm mean cumulative rainfall, Supplementary Fig. S1b). Both meteorological stations showed that rainfall events over 2019-2021 were more concentrated in the autumn season compared to the standard reference period, particularly in November (mean cumulative rainfall over 2019–2021: 128 mm and 150 mm for La Chiappa and Cap Pertusato stations respectively; Supplementary Fig. S1b). The description of the meteorological context over the study period at monthly scale can be found in the Supplementary Material.

**Fig. 3:**
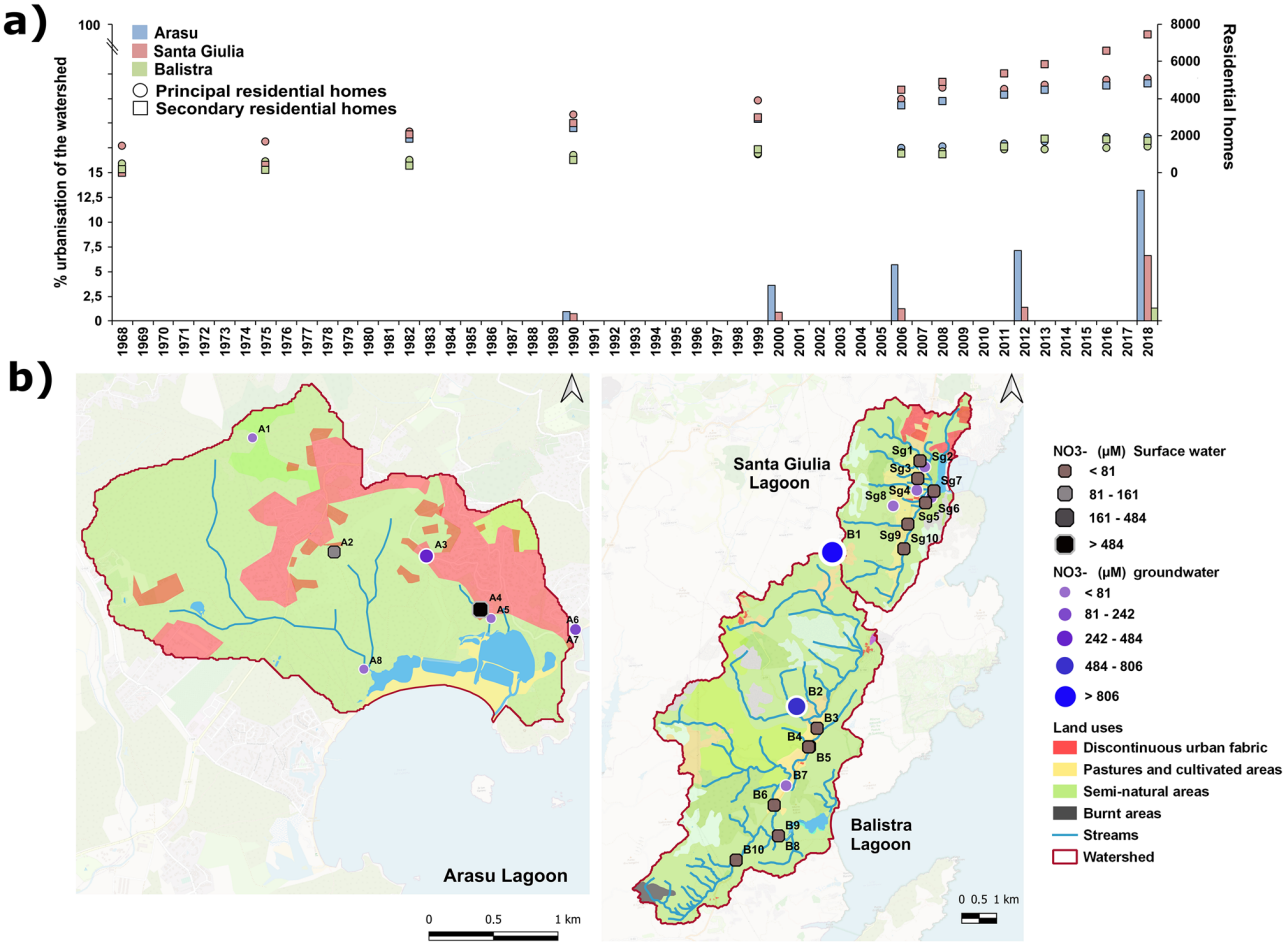
Representation of watershed characteristics: **a** urbanisation increase is represented as the increase in principle or secondary residential homes over the 1968–2018 period (right scale, points) and as the percentage of the watershed’s urbanised area (left scale, bars); **b** land uses (2018) are shown, together with sampling points for the nitrates (point size represents concentrations, while point shape indicates weather the sample is from surface water (squares) or groundwater (rounds))

As for the quantification of the urbanisation rate of the three watersheds, a dramatic growth of residential homes over the surroundings of all three lagoons emerged from 1968 to 2018 (Fig. 3a). Principal residential homes tripled for all municipalities in the three considered areas (Fig. 3a). A greater proportional rise was highlighted for secondary residential homes. They increased eight-fold for the vicinity of Balistra lagoon, while they experienced a thirty- and three-hundred-fold increase for the surroundings of Arasu and Santa Giulia lagoons, respectively (Fig. 3a). Overall, the highest urbanisation rate over the last 30 years was observed for the Arasu lagoon watershed, which increased from 0.96% in 1990 to 13.2% in 2018 (Fig. 3a), while the Santa Giulia lagoon watershed experienced an increase of around 6% in urbanisation over the same period (Fig. 3a) and the progression of urbanisation for Balistra lagoon was negligible (Fig. 3a). From land use analysis, all three watersheds were predominantly composed of natural or semi-natural zones, the Balistra lagoon watershed being almost exclusively natural, at 93% land cover (Fig. 3b). The Arasu lagoon watershed appears at present as the most urbanised, with 13.2% of urban land cover in 2018, compared to 6.7% and 1.3% at Santa Giulia and Balistra, respectively (Fig. 3a, b). Rural urbanisation is concentrated in the north and north-eastern part of the basin for Arasu lagoon.

Sampling points in the urbanised area of the Arasu lagoon watershed showed NO_3_^− ^concentrations ranging between 164.5 and 487.1 µM for surface waters and between 171.0 and 469.4 µM for groundwater (Fig. 3b). All the sampling points, except one, were situated on the flow line of surface and ground waters to the eastern basin of Arasu lagoon (Fig. 3b).

The Santa Giulia lagoon watershed presents the most urbanised zone in the north part and upstream of the basin. Also, 19% of the watershed is constituted by agricultural areas, located mainly in the western part (Fig. 3b). Highest values of NO_3_^−^ were recorded in stations downstream of the agricultural areas ranging between 83.9 µM in groundwater and 30.7 µM in a temporary river (Fig. 3b). Those NO_3_^−^ concentrations are very close to the natural regional baseline level (i.e. < 113 µM). The Balistra lagoon watershed contains no urbanisation and only very little agricultural development. However, it showed the highest values of NO_3_^−^ in groundwater, with occasional peaks of 837.1 and 1269.4 µM at two sampling sites upstream of the basin, far from the lagoon (3 and 7 km respectively), in hamlets without public sanitation (Fig. 3b). These were the only two samples to exceed the World Health Organisation drinking water threshold for nitrate of 50 mg L^−1^, the equivalent of 806 µM (World Health Organization 2011).

#### Ecological Functioning – Environmental Variables

During the study, no notable meteorological events preceded sampling days. The only remarkable feature was a general greater exposure to north-easterly winds of Santa Giulia lagoon and sometimes to westerly winds of Balistra lagoon, especially in 2020.

For all three lagoons, water temperatures followed the seasonal norms and ANOVA detected a strong effect of season (*p* < 0.001 for all; Arasu: *F*_3,12_ = 78.960, *ω*_p_^2^ = 0.907; Santa Giulia: *F*_3,8_ = 30.407, *ω*_p_^2^ = 0.846; Balistra: *F*_3,8_ = 53.785, *ω*_p_^2^ = 0.908; Supplementary Fig. S2). Oxygen saturation never fell below 88% at Balistra lagoon, while minimum values of 58% and 60% were found at Arasu (summer 2021, St2) and Santa Giulia (summer 2020, St2), respectively (Supplementary Fig. S2). A significant seasonal effect was found from the ANOVA test for Balistra lagoon only (*p* = 0.015; Supplementary Fig. S2). Santa Giulia was overall the most turbid lagoon, with mean turbidity values of 10.6 FNU and reaching a maximum of 42 FNU in summer 2020 at St2, against mean turbidity values of 0.6 and 1.1 FNU for Arasu and Balistra, respectively (Supplementary Fig. S2). This parameter showed significant spatial difference in Arasu lagoon (K-W: *H* = 0.191, *n* = 24, *p* = 0.049; Dunn’s test: St1 ≠ St2 = St3).

**Fig. 4:**
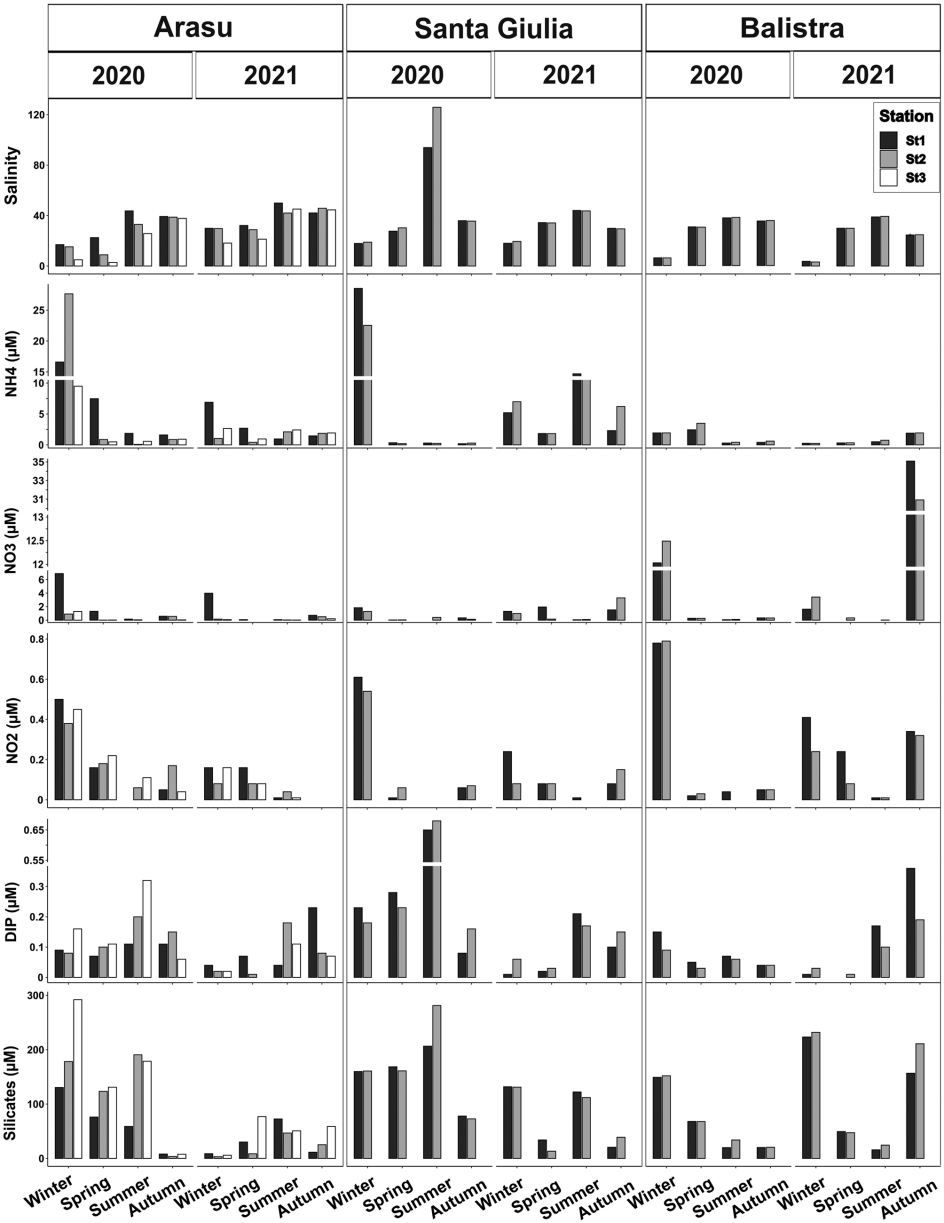
Environmental variables measured for the three lagoons in the two sampling years 2020–2021. Salinity (with error bars representing Standard Error over the three replicates measured in situ) and nutrients concentrations (NH_4_^+^, NO_3_^**−**^, NO_2_^**−**^, DIP, Silicates; µM) for the water basins are shown by sampling stations

Salinity ranged between 2.8 (spring 2020, St3) and 49.9 (summer 2021, St1) in Arasu lagoon with lowest values systematically registered at St2 or St3, except for autumn 2021 (Fig. 4). Santa Giulia lagoon showed mean salinity of 40, with a peak at 125.9 in summer 2020 at St2 (Fig. 4). Balistra lagoon showed internal spatial homogeneity, with minimum values during the winters of both years considered (min.: 3.2, winter 2021, St2) and mean overall value of 26.1 (Fig. 4). An important seasonal effect was detected from the ANOVA test for all three lagoons (Arasu: *F*_3,12_ = 5.356, *ω*_p_^2^ = 0.353, *p* = 0.014; Santa Giulia: *F*_3,8_ = 9.993, *ω*_p_^2^ = 0.628, *p* = 0.004; Balistra: *F*_3,8_ = 72.827, *ω*_p_^2^ = 0.931, *p* < 0.001).

Highest nutrient values were found during winters for all lagoons. Santa Giulia showed highest mean values for NH_4_^+^, DIP and silicate concentrations (6.5, 0.2 and 118.3 µM respectively), while highest mean values for NO_2_^−^ and NO_3_^−^ concentrations were registered in Balistra lagoon at 0.2 and 5.6 µM, respectively. At Arasu lagoon, ANOVA detected a moderate effect of the season for NH_4_^+^ (*F*_3,12_ = 4.777, *ω*_p_^2^ = 0.321, *p* = 0.020), resulting in highest values in winter, compared to other seasons according to the Tukey test. In addition, effects of season and station were found for NO_3_^−^ (K-W: *H* = 0.365, *n* = 24, *p* = 0.016 (season) and *H* = 0.236, *n* = 24, *p* = 0.031 (station)), indicating significant differences of winter from spring and summer seasons and highest values at St1 compared to the other stations (Fig. 4).

NO_2_^−^ differed among seasons according to the ANOVA test for all three lagoons (Arasu: *F*_3,12_ = 7.427, *ω*_p_^2^ = 0.445, *p* = 0.005; Santa Giulia: *F*_3,8_ = 4.916, *ω*_p_^2^ = 0.423, *p* = 0.032; Balistra: *F*_3,8_ = 6.772, *ω*_p_^2^ = 0.520, *p* = 0.014). A similar result was found for silicate concentrations, except in Balistra lagoon (ANOVA *F*_3,8_ = 4.586, *ω*_p_^2^ = 0.402, *p* = 0.038) where they seemed to follow a seasonal decreasing gradient from winter to summer (Fig. 4).

**Fig. 5:**
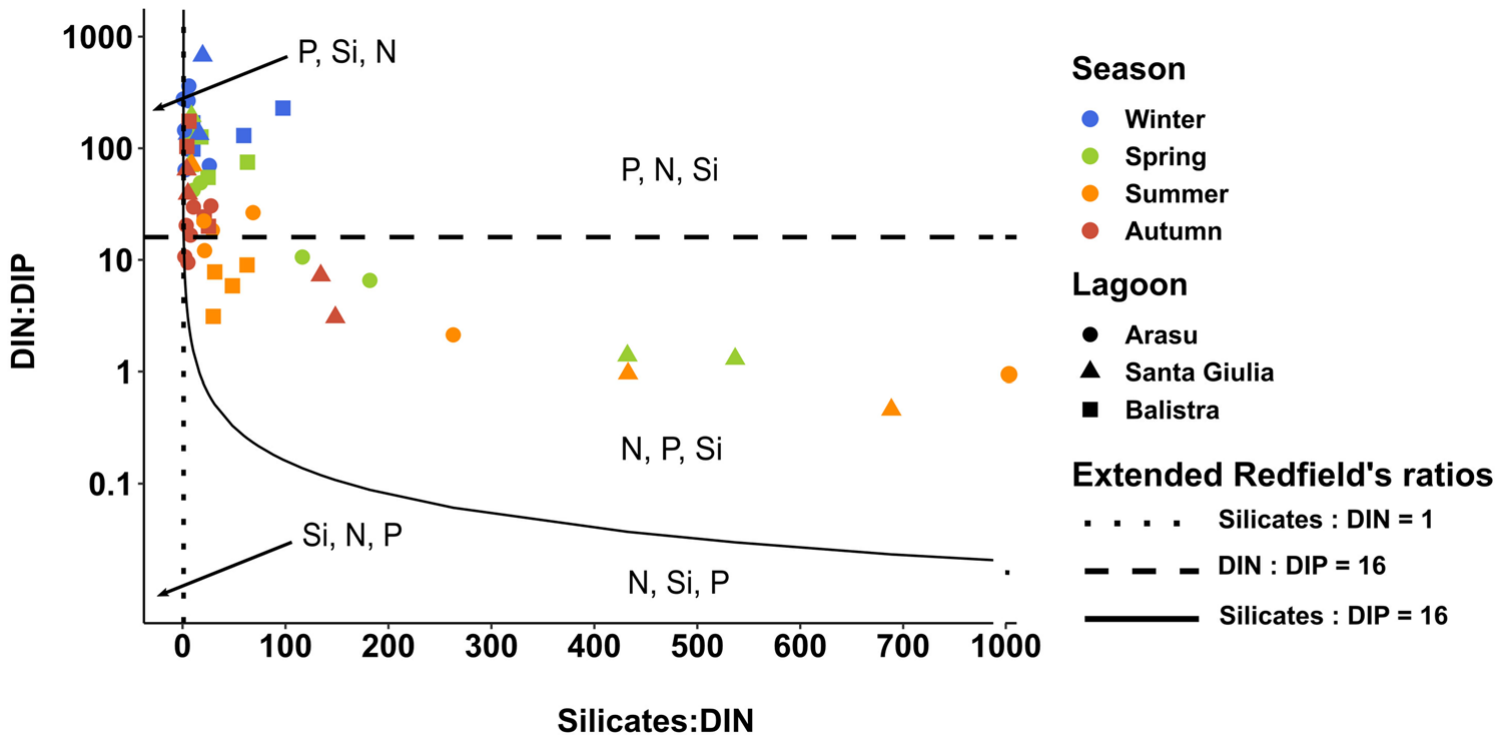
Molar inorganic nutrients ratios measured in the three lagoons at different seasonal samplings. Lines represent molar ratios defined by Redfield et al. (1963) and Brzezinski (1985), with Silicates:DIN = 1, DIN:DIP = 16, and Silicates:DIP = 16, and in the delimited areas nutrients are given in their potential limitation’s priority order (Rocha et al. 2002)

ANOVA tests on the ratio between total dissolved inorganic nitrogen (DIN, calculated as NH_4_^+^ + NO_2_^−^ + NO_3_^−^) and DIP (DIN/DIP, data not shown) revealed significant differences in N inputs and consequent nutrients imbalance. Results showed an effect of season factor for Arasu and Balistra lagoons (ANOVA *F*_3,11_ = 11.475, *ω*_p_^2^ = 0.577, *p* = 0.001 and* F*_*3*,7_ = 12.662, *ω*_p_^2^ = 0.700, p = 0.003, respectively), but different trends for the two cases: for Arasu the ratio was higher in winter than other seasons, while for Balistra summer showed a lower value. To characterise the lagoons’ trophic status, nutrients ratios were compared to Redfield et al. 1963 and Brzezinski 1985 ratios (Fig. 5). Overall, DIP proved to be the most limiting factor, but Santa Giulia lagoon and summer season were sometimes DIN-limited, while silicates limitation was never observed (Fig. 5).

**Fig. 6:**
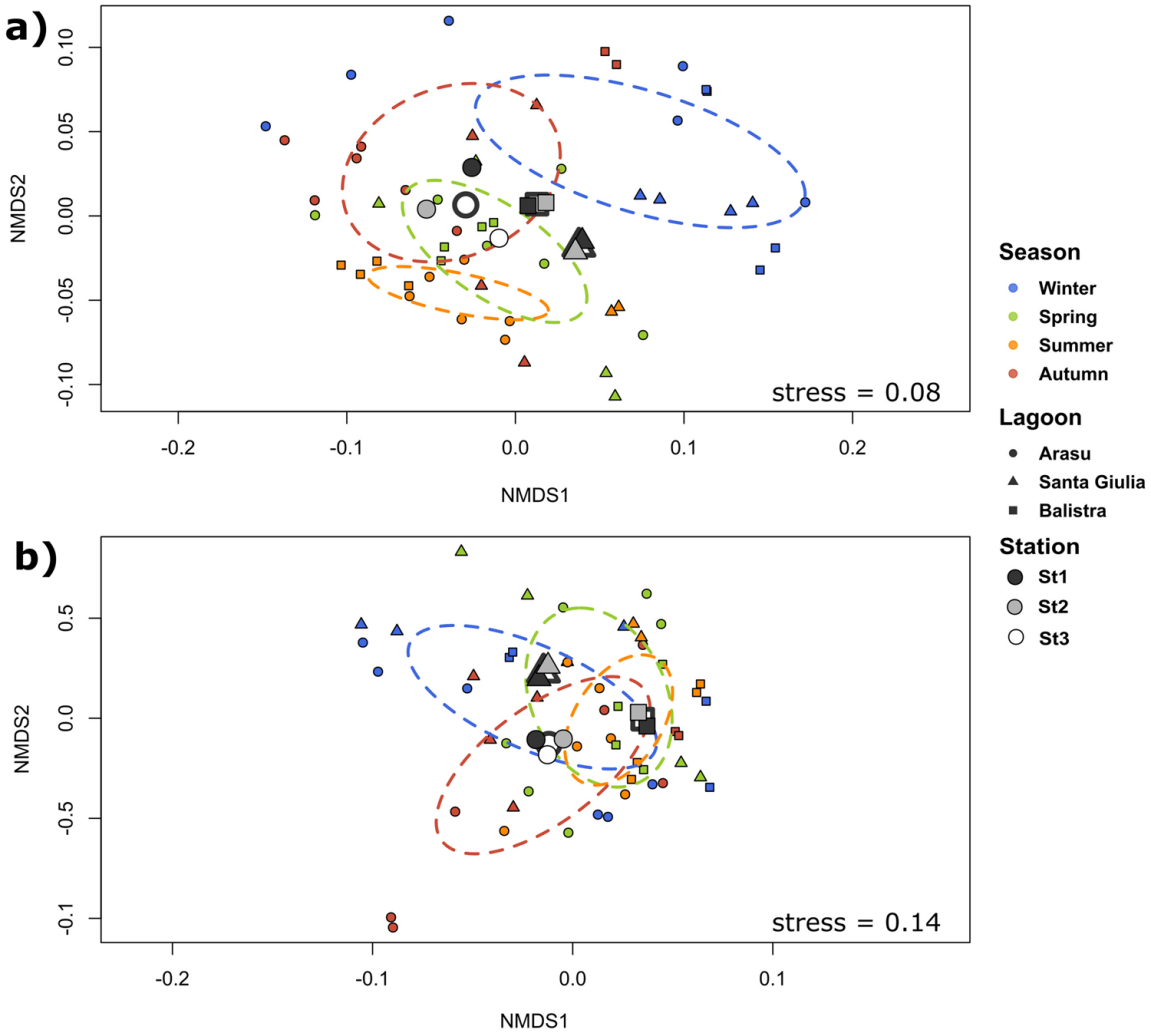
NMDS representation of total dataset for **a** abiotic and **b** biotic variables. Samples are color-coded for seasons, shapes indicate lagoons and stations. Ellipses are shown to describe seasonal clustering, together with lagoon and station centroids

In order to obtain an overall characterisation of the three ecosystems, a comprehensive analysis was performed on abiotic parameters datasets to detect potential differences between lagoons and eventually their seasonal and spatial patterns. The analysis highlighted significant effects of both the season and the lagoon factors. From NMDS visualisation, seasons resulted clustered (Fig. 6a). Lagoon centroids were clearly separated and station centroids within lagoons only showed high dispersion for Arasu lagoon (Fig. 6a). The PERMANOVA test highlighted moderate effects of season (*R*^2^ = 0.308, *ω*_p_^2^ = 0.305, *p* = 0.001) and lagoon (*R*^2^ = 0.150, *ω*_p_^2^ = 0.169, *p* = 0.001). All seasons were different from each other according to the pairwise test, while Santa Giulia was different from Arasu (*R*^2^ = 0.155, *p* = 0.003) and Balistra (*R*^2^ = 0.144, *p* = 0.011) lagoons, which did not differ from each other (*R*^2^ = 0.057, *p* = 0.099).

#### Ecological Functioning – Biotic Variables

**Fig. 7:**
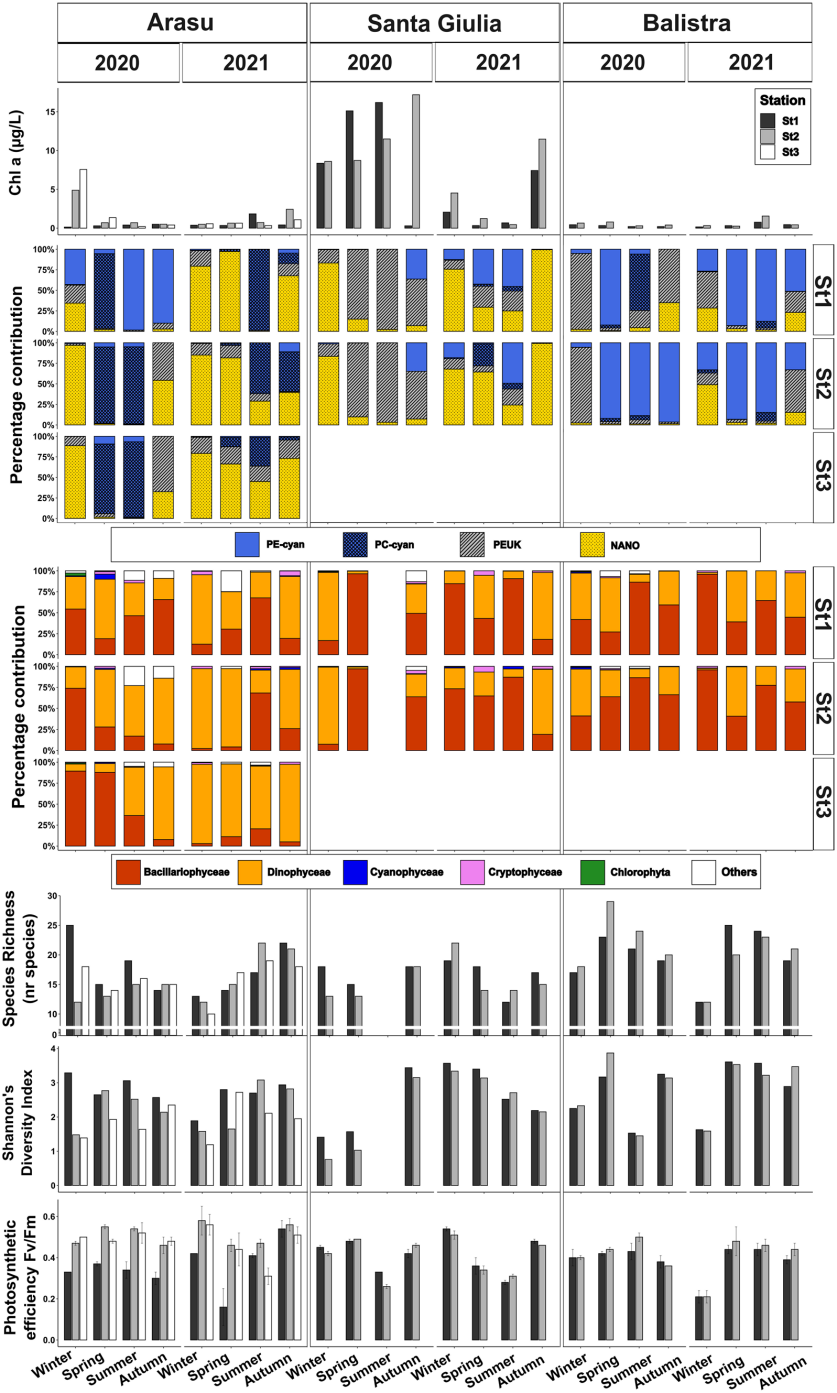
Biotic parameters considered for the three lagoons in the two sampling years 2020–2021: chlorophyll *a*, pico- and nano-phytoplankton, microphytoplankton class composition, species richness, Shannon’s Diversity Index and photosynthetic efficiency (Fv/Fm) of the assemblages are represented

In general, Santa Giulia lagoon stood out for its Chl *a* content, with a mean of 7.1 µg L^−1^ reaching a maximum of 17.2 µg L^−1^ in autumn 2020 at St2 (Fig. 7). Arasu lagoon showed Chl *a* values ranging from 0.1 to 7.6 µg L^−1^, usually lower at St1, and mean value of 1.1 µg L^−1^ (Fig. 7), while in Balistra lagoon Chl* a* content never exceeded 1.6 µg L^−1^, with a mean of 0.5 µg L^−1^, and did not display marked differences between stations (Fig. 7).

Overall, differences between seasons or stations within lagoons were less marked. No significant spatial differences were found for the pico-, nano- and micro-phytoplankton communities’ compositions. No marked differences could be seen between stations for communities’ percentage compositions: Santa Giulia and Arasu were mostly dominated by PEUK and NANO, except for an almost complete dominance of picocyanobacteria in Arasu lagoon in spring and summer at all stations and in autumn at St1 in 2020 (Fig. 7). NANO contribution in Balistra lagoon was limited, as the community was systematically dominated by picophytoplankton and mostly PE-cyan (Fig. 7). Diatoms constituted almost systematically the major component of microphytoplankton assemblages, regardless of season and lagoon, except for St2 and St3 in Arasu lagoon, which were predominantly dominated by Dinoflagellates (Fig. 7). Yet no significant effect of station could be highlighted for microphytoplankton composition. A moderate seasonal effect was only found for the Diatoms group in Balistra lagoon (ANOVA *F*_3,8_ = 10.446, *ω*_p_^2^ = 0.639, *p* = 0.004), with highest contributions in summer and autumn compared to spring (Fig. 7).

Only station effects were detected for Arasu lagoon for Shannon’s Diversity Index and Fv/Fm (ANOVA *F*_2,12_ = 6.950, *ω*_p_^2^ = 0.331,* p* = 0.010 and *F*_2,12_ = 8.917, *ω*_p_^2^ = 0.397, *p* = 0.004 respectively), indicating St1 differed from the others according to the Tukey test. In particular, St1 repeatedly showed higher diversity but lower photosynthetic efficiency of the community (Fig. 7). Finally, a moderate effect of season in Santa Giulia lagoon was identified for Fv/Fm (ANOVA *F*_3,8_ = 6.499, *ω*_p_^2^ = 0.508, *p* = 0.015), signifying a difference of summer season (lower values, Fig. 7) compared to the others, according to the Tukey test.

Overall seasonal and spatial patterns between and within lagoons were investigated through a comprehensive analysis on overall biotic variables. Significant effects of both the season and the lagoon factors were detected. In the NMDS visualisation of biotic variables, season clusters were superposed (Fig. 6b). Lagoon centroids were well distinct, with less station dispersion compared to abiotic variables NMDS analysis (Fig. 6b). An effect of season (*R*^2^ = 0.154, *ω*_p_^2^ = 0.109, *p* = 0.003) and lagoon (*R*^2^ = 0. 135, *ω*_p_^2^ = 0.106, *p* = 0.001) emerged from PERMANOVA, and according to the pairwise test all lagoons differed from each other. Conversely, no clear distinction could be highlighted for seasons, except summer, which differed from winter (*R*^2^ = 0.159, *p* = 0.018) and autumn (*R*^2^ = 0.188, *p* = 0.018) according to the pairwise test.

A complete list of microphytoplankton taxonomic units found at each lagoon and season is reported in Table S1 (Supplementary). A closer look at the microphytoplankton community structure and composition revealed that total cellular abundance was generally higher at Arasu and Santa Giulia lagoons than Balistra lagoon, almost systematically exceeding 1x10^6^ cell L^−1^ (Table 1). In Balistra lagoon, these magnitudes were only reached in winter and spring 2020 and in summer of both years (Table 1). Arasu and Santa Giulia lagoons’ communities were frequently dominated by one or a few taxa at high densities, frequently blooming (cellular density > 100,000 cell L^−1^; Belin and Neaud-Masson 2017), as also confirmed by lower species richness and Shannon’s Diversity Index (Table 1, Fig. 7). In Balistra lagoon, blooms were rare: the only bloom was recorded during summer in 2020 and was constituted by the diatom *Chaetoceros* spp. (Table 1). Except this season, Balistra lagoon always showed the highest diversity and species richness amongst the lagoons (Fig. 7). In any case, sometimes dinoflagellates were also present in high densities or even dominant, at times with potentially harmful species, such as *Kryptoperidinium foliaceum*, reaching 72×10^3^ cell L^−1^ in winter 2020 (St2), *Prorocentrum micans* in spring and autumn 2021 (reaching up to 24×10^3^ cell L^−1^ in autumn 2021, St1) and *Protoperidinium* sp. occurring occasionally (Table 1). In Arasu lagoon, differences between stations could be observed: total abundances were generally higher at St2 and St3 than St1, which also showed lower dinoflagellate contribution compared to the other two stations (Table 1, Fig. 7). The presence or dominance of dinoflagellates was almost systematic and frequent blooms of dinoflagellate species, sometimes potentially toxic, occurred (Table 1). For instance, *Prorocentrum cordatum* produced an extended bloom over St2 and St3 and was dominant all over the basin in winter 2020 (Table 1); in addition, *P. micans* bloomed frequently and persisted over the basin and across seasons and years, reaching a peak at 518×10^3^ cell L×^1^ in spring 2021 at St3 (Table 1). Potentially toxic *Akashiwo sanguinea* was sporadically present and bloomed at 250×10^3^ cell L^−1^ in spring 2021, while dominance of *Chaetoceros* spp. was limited to St3 (Table 1). In Santa Giulia lagoon the two stations were quite similar: potentially toxic or non-toxic dinoflagellates often dominated the community or bloomed, especially during autumn. For instance, *P. cordatum* reached 68×10^3^ cell L^−1^ in autumn 2020 at St2 and *P. micans* bloomed in autumn 2021 at St1 reaching 102×10^3^ cell L^−1^ (Table 1). In winter 2020 the lagoon experienced a high bloom of *Diplopsalis* sp., reaching 1×10^7^ cell L^−1^ magnitude at both stations, a scale that was reached again only by *Chaetoceros* spp. in spring 2020, corresponding in both cases to lowest diversity and species richness values (Table 1, Fig. 7).

**Table 1:** Summary description of dominant microphytoplankton taxa, with percentage proportion in terms of abundance relative to total cellular abundance

		***Arasu***						***Santa Giulia***				***Balistra***			
		***St1***		***St2***		***St3***		***St1***		***St2***		***St1***		***St2***	
		**Dominant Taxon and percentage proportion**	**Total abundance** × **10**^**3**^** cell L**^**−1**^	**Dominant Taxon and percentage proportion**	**Total abundance** × **10**^**3**^** cell L**^**−1**^	**Dominant Taxon and percentage proportion**	**Total abundance** × **10**^**3**^** cell L**^**−1**^	**Dominant Taxon and percentage proportion**	**Total abundance** × **10**^**3**^** cell L**^**−1**^	**Dominant Taxon and percentage proportion**	**Total abundance** × **10**^**3**^** cell L**^**−1**^	**Dominant Taxon and percentage proportion**	**Total abundance** × **10**^**3**^** cell L**^**−1**^	**Dominant Taxon and percentage proportion**	**Total abundance** × **10**^**3**^** cell L**^**−1**^
**2020**	Winter	Undetermined diatom (22%)	81	Undetermined diatom (69%)	**1134**	Undetermined diatom (79%)	**3157**	*Diplopsalis* sp. (80%)	**4097**	*Diplopsalis* sp. (90%)	**1116**	*Kryptoperidinium foliaceum (44%)*	**155**	*Kryptoperidinium foliaceum (45%)*	**161**
		*Prorocentrum cordatum* (20%)		*Prorocentrum cordatum* (15%)		*Prorocentrum cordatum* (5%)		*Navicula* spp. (5%)				Fragilariophycideae (33%)		Fragilariophycideae (32%)	
		*Amphora* sp. (17%)		*Prorocentrum micans* (9%)		*Chaetoceros* spp. (4%)		Undetermined diatom (3%)							
	Spring	Undetermined dinoflagellate (40%)	**302**	*Prorocentrum micans* (37%)	**782**	*Chaetoceros* spp. (62%)	**1049**	*Chaetoceros* spp. (73%)	**6857**	*Chaetoceros* spp. (85%)	**2648**	Undetermined dinoflagellate (35%)	56	Undetermined dinoflagellate (20%)	**101**
		*Prorocentrum micans* (26%)		*Prorocentrum cordatum* (17%)		Small centric diatoms (17%)		Small pennate diatoms (11%)		Small pennate diatoms (5%)		*Scrippsiella trocoïdea (21%)*		Small pennate diatoms (16%)	
								*Nitzschia* sp. (7%)							
	Summer	*Amphora* sp. (32%)	**225**	*Prorocentrum micans* (41%)	**468**	Undetermined dinoflagellate (56%)	**243**					*Chaetoceros* spp. (78%)	**162**	*Chaetoceros* spp. (80%)	**263**
		Undetermined dinoflagellate (21%)		Undetermined (23%)		*Chaetoceros* spp. (34%)									
	Autumn	*Amphora* sp. (45%)	**177**	*Prorocentrum micans* (63%)	**171**	*Prorocentrum micans* (41%)	**282**	*Prorocentrum cordatum* (22%)	**128**	*Amphora* sp. (33%)	**354**	*Protoperidinium* sp. (24%)	32	*Protoperidinium* sp. (23%)	36
								*Navicula* spp. (17%)		*Prorocentrum cordatum* (19%)		*Cocconeis* sp. (17%)		*Cocconeis* sp. (23%)	
**2021**	Winter	*Protoperidinium* sp. (60%)	**208**	*Prorocentrum micans* (73%)	**209**	*Prorocentrum micans* (80%)	**194**	*Navicula* spp. (25%)	**173**	*Navicula* spp. (30%)	**126**	Fragilariophycideae (70%)	42	Fragilariophycideae (70%)	41
		*Prorocentrum micans* (19%)		*Protoperidinium* sp. (11%)				Fragilariophycideae (14%)		*Alexandrium* spp. (15%)					
		*Amphora* sp. (10%)		*Akashiwo sanguinea* (10%)				*Cocconeis* sp. (14%)		Fragilariophycideae (11%)					
	Spring	*Amphora* sp. (25%)	84	*Prorocentrum micans* (73%)	**147**	*Prorocentrum micans* (44%)	**1189**	*Alexandrium* spp. (26%)	53	*Nitzschia* sp. (21%)	47	*Prorocentrum micans (20%)*	60	*Prorocentrum micans (20%)*	67
		Others (25%)				*Akashiwo sanguinea* (21%)		Fragilariophycideae (17%)		Fragilariophycideae (18%)		*Protoperidinium sp. (15%)*		*Protoperidinium* sp. (16%)	
		*Prorocentrum micans* (18%)				*Kryptoperidinium foliaceum* (8%)									
	Summer	*Amphora* sp. (36%)	**494**	*Nitzschia* sp. (41%)	**124**	*Prorocentrum micans* (65%)	**106**	*Nitzschia* sp. (46%)	**129**	*Nitzschia* sp. (44%)	**170**	*Nitzschia* sp. (24%)	**106**	*Chaetoceros* spp. (29%)	**104**
				*Prorocentrum micans* (13%)				*Navicula* spp. (13%)		*Navicula* spp. (15%)		*Chaetoceros* spp. (16%)		*Nitzschia* sp. (29%)	
												*Alexandrium* spp. (12%)			
	Autumn	*Prorocentrum micans* (47%)	76	*Prorocentrum micans* (49%)	**251**	*Prorocentrum micans* (67%)	**166**	*Prorocentrum micans* (60%)	**171**	*Prorocentrum micans* (65%)	99	*Prorocentrum micans (40%)*	61	*Prorocentrum micans (26%)*	44
						*Akashiwo sanguinea* (14%)		Fragilariophycideae (11%)				Fragilariophycideae (22%)		*Chaetoceros* spp. (18%)	
								*Akashiwo sanguinea* (10%)							

Abundance values in bold represent densities over bloom threshold (> 100,000 cell L^−1^)

At lagoon scale, some interesting significant correlations emerged. Overall, Diatoms were not correlated with indexes of richness, diversity or photosynthetic activity, but Dinoflagellates were negatively correlated with Shannon’s Diversity Index both in Arasu (*r* = −0.41, *n* = 24, *p* = 0.046) and Santa Giulia (*r* = -0.61, *n* = 14, *p* = 0.022) lagoons. They were also positively correlated with Fv/Fm in Arasu (*r* = 0.48, *n* = 24, *p* = 0.017) and Santa Giulia (*r* = 0.62, *n* = 14, *p* = 0.017,) lagoons together with Chl *a* (Arasu: *r* = 0.51, *n* = 24, *p* = 0.010; Santa Giulia: *r* = 0.70, *n* = 14, *p* = 0.006). In Santa Giulia lagoon Shannon’s Diversity Index was also negatively correlated with Chl *a* (*r* = −0.58, *n* = 14, *p* = 0.030), while Fv/Fm was negatively correlated with salinity (*r* = −0.63, *n* = 14, *p* = 0.015), water temperature (*r* = −0.60, *n* = 14, *p* = 0.025) and PC-cyan (*r* = −0.58, *n* = 14, *p* = 0.029). Balistra lagoon showed a completely opposite mode of functioning, with a positive correlation between Chl *a* and Shannon’s Diversity Index (*r* = 0.54, *n* = 14, *p* = 0.045), and between Fv/Fm and salinity (*r* = 0.67, *n* = 14, *p* = 0.009), water temperature (*r* = 0.68, *n* = 14, *p* = 0.007), PC-cyan (*r* = 0.54, *n* = 14, *p* = 0.044) and PE-cyan (*r* = 0.67, *n* = 14, *p* = 0.009).

## Discussion

### Ecological Functioning of the Three Small Lagoons

Despite some small local differences potentially affecting the three lagoons’ hydrological functioning at small scale, we highlighted evidence of climate change such as the extension of the dry period, the occurrence of drought outside the summer season and the intensification of the rainy period over shorter periods. Such impacts are exemplified by the small size of the studied sites that function as temporary coastal lagoons *sensu* Latron et al. (2022). We also established that our case study lagoons have been subjected to increasing rural urbanisation over the past few decades and especially seasonal pressure linked to summer tourism. In fact, at regional level, according to available data from the ^©^Insee (https://www.insee.fr), during the 1999–2021 period the resident population increased linearly by a 1.3% annual rate, with the year 2021 reaching an increase of 33% compared to 1999. Furthermore, over the same time span, the number of summer travellers increased by a 0.8% annual rate, with a peak in 2018 at 23% more travellers than the average over the entire period (DREAL Corse). The most directly impacted lagoons appear to be Arasu and Santa Giulia with a 12% and 6% urbanisation rate increase of their watersheds, respectively, within the last 30 years. Regarding our indicator for anthropogenic pollution, the nitrates, the natural baseline concentration for groundwater is estimated at between 5 and 7 mg L^−1^ maximum (Appelo and Postma 2005), the equivalent of 81 and 113 µM, hence nitrate concentrations exceeding this threshold can be related to human activities impacting groundwater quality.

**Fig. 8:**
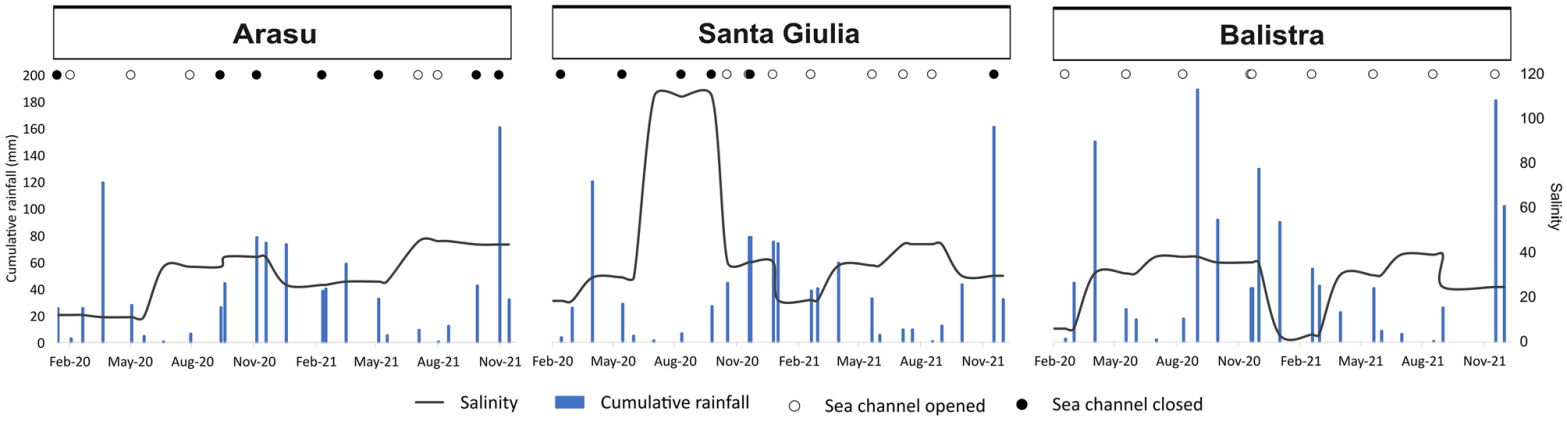
Representation of daily rainfall (mm, scale on the left), mean salinity (scale on the right) over each entire water basin and status (opened as white or closed as black points at the top of the graph) of the sea channel outlet, based on available dates over the study period

**Fig. 9:**
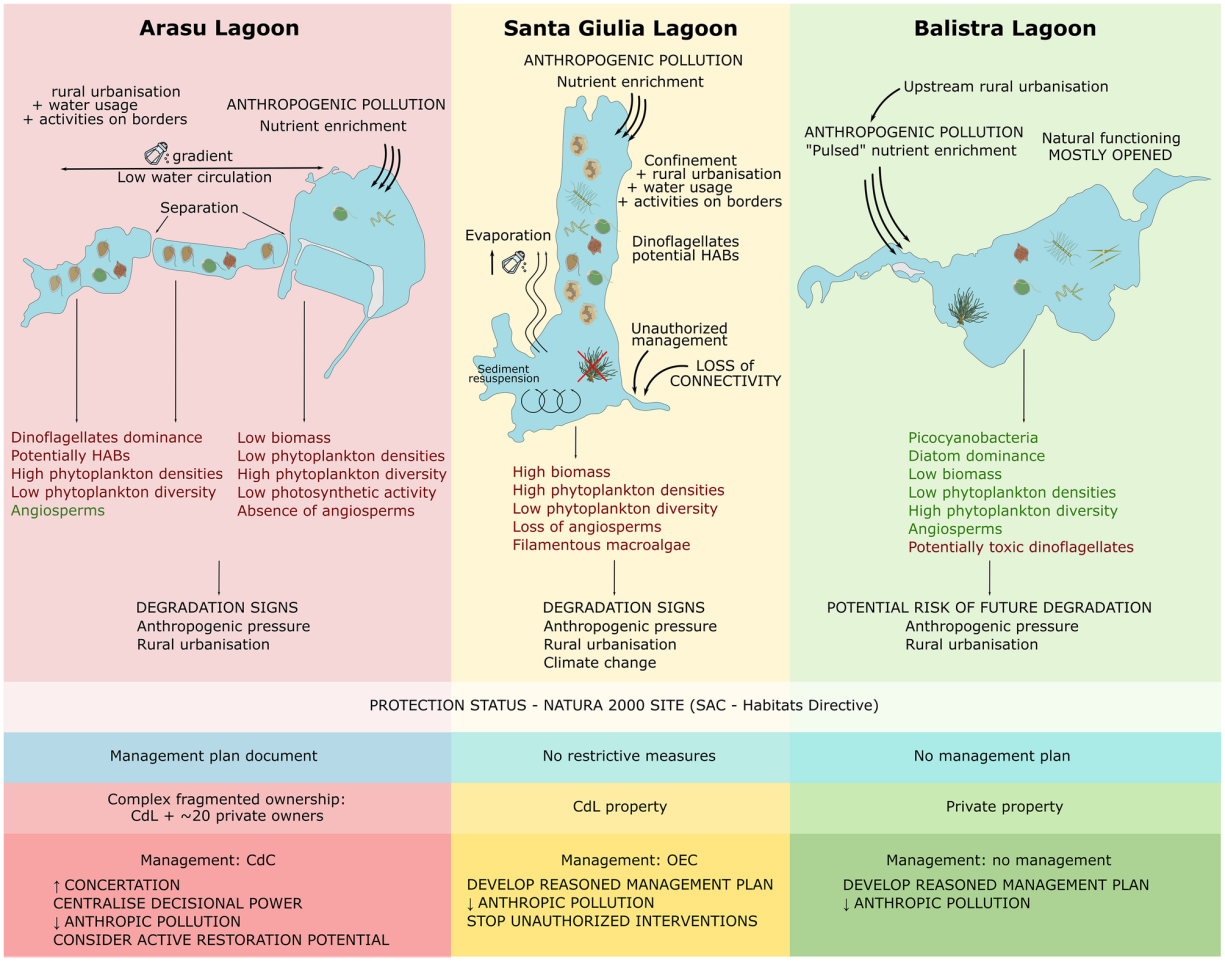
Schematic representation of the main features of the dynamics, functioning, pressures and management of the three lagoons in their current anthropogenic contexts

For Arasu, a small aquifer is present in the western part of its watershed. Also, in the north-eastern part, there is a permanent stream with its accompanying groundwater. Since the stream is located in the most densely urbanised area of the watershed, it has a potential role in influencing anthropogenic pollution inflow to the lagoon. High NO_3_^−^ concentrations seemed to be attributable to sanitation defects inducing wastewater leakages to the groundwater. Salinity variations were clearly linked to the opening status of the sea channel outlet (Fig. 8). The lagoon functioning is marked by the major anthropogenic alterations to which it was subjected in the past, i.e. the construction of roads on dams dividing the basin into three separate portions. There is a sort of lagged response of salinity to rainfall events and sea channel opening times since freshwater and marine inputs are located at different and physically separate sites. In particular, fluctuations in salinity and nutrient concentrations were markers of the limited water circulation between the three separate basins (Fig. 9). The eastern basin was the most impacted by urbanisation and the least productive in terms of phytoplankton biomass, showing signs of degradation, including the complete absence of angiosperm meadows (Fig. 9). Lower phytoplankton biomass might be a consequence of greater connection with the sea, where biomass is generally low (Sarno et al. 1993). Another cause might be anthropogenic impact, which is higher in this area, linked to the exposure to some kind of chemicals or pollution (Lafabrie et al. 2013 and references herein). We hypothesised some sort of current or legacy pollution might be responsible for the degradation. For instance, Lafabrie et al. (2013) demonstrated that sediments can be a sink for contaminants and affect the phytoplankton community by long-term exposure from sediment resuspension. Moreover, absence of angiosperms might be due to eutrophication, pollutants or legacy effects of past sediment rearrangement*,* as these factors are known to cause a decline in angiosperm coverage in disturbed lagoon systems (Sfriso et al. 2017, 2019, 2021). The high dominance of potentially harmful dinoflagellates, blooms and low species diversity, especially at the St2 and St3 basins, indicate degradation of the lagoon, probably due to confinement (Sarno et al. 1993). *Prorocentrum micans* in particular produced intense and very long-lasting blooms expanding to all three basins. Potentially toxic *Prorocentrum cordatum* and *Akashiwo sanguinea* also sometimes bloomed. These potentially toxic species are known to produce high density blooms, especially during summer/late autumn, linked to confinement and nutrient inputs (Sarno et al. 1993; Sahraoui et al. 2013; Bouchouicha Smida et al. 2014).

The functioning of Santa Giulia lagoon proved to be an excellent example of the impact of human interventions and the importance of the connection with the sea (Fig. 9). The sea outlet tends to close naturally due to the accumulation of *Posidonia oceanica* litter. The re-establishment of the connection with the sea seldom takes place naturally. This has already led to some strong evaporation during summer and partial drying of some areas of the basin in the past. At present, most of the time business owners on the adjacent beach privately undertake the artificial opening of the sea channel, without official regulation, in order to maintain good water circulation and prevent unpleasant odours (Fig. 9). In 2020, in addition to low rainfall events, tourism activities were negatively impacted due to the Covid-19 health crisis (lockdowns, restrictions for travel, etc.), with a record loss of 31% of travellers compared to the mean over the 1999-2021 period (DREAL Corse). Artificial openings of the sea outlet were not carried out as often as usual, so that it stayed obstructed by litter most of the time, ultimately leading to the almost complete drying-up of the water basin during summer (Figs. 8 and 9). This had catastrophic consequences for aquatic biotic community, by causing fish mortality, phytoplankton biomass development but depletion of photosynthetic efficiency, as well as a complete loss of angiosperm meadows and the development of filamentous macroalgal communities (Fig. 9). Loss of connectivity with the sea has already been associated with the increase of eutrophication and phytoplankton biomass in lagoon environment, and our study case confirms the same pattern for small sized lagoons (Pereira Coutinho et al. 2012; Jones et al. 2018). Moreover, high phytoplankton biomass was negatively correlated with diversity, meaning the community was frequently dominated by one or a few taxa and blooms were recurrent. Blooms were alternatively due to diatoms and dinoflagellates, but from autumn 2020 diversity increased, while biomass decreased. This could be a consequence of the drought episode that occurred during summer 2020; in fact, it has already been observed that desiccation can boost phytoplankton diversity (Rojo et al. 2012). Dryness increases eutrophication by releasing nutrients and can structure diversity by favouring the development of stress-tolerant species. In the short term, temporary isolation and the appearance of disconnected sites can promote heterogeneity and consequently enhance species richness (Angeler et al. 2010; Rojo et al. 2012). Extreme situations inducing stress could promote the development of potentially harmful species. For instance, the dinoflagellate *Prorocentrum cordatum*, and *Prorocentrum* spp. in general, is a stress-tolerant potentially harmful taxon and is especially resistant towards high salinity (Sahraoui et al. 2013). This species appeared at Santa Giulia lagoon in autumn, after the summer salinity stress, and *Prorocentrum* sp. was sporadically present at high densities in the lagoon over the sampling period and occasionally blooming. The majority of taxa recorded in Santa Giulia lagoon, such as *Amphora* sp., *Nitzschia* spp. or *Cocconeis* sp., are typically tychoplanktonic, meaning they are generally found attached to substrate and only occasionally in the water column, following some physical process. This is linked to the shallowness of the basin (Carić et al. 2011), which could also explain the high turbidity found at this site (Fig. 9): the water body is often exposed to wind so due to its shallowness sediment is easily resuspended, the water column is well mixed and may result in an increase in Chl *a* concentration with the importation of benthic species (Péquin et al. 2017; Trombetta et al. 2021).

Balistra lagoon is an example of the need to consider lagoon functioning together with the watershed influence. Despite its supposedly pristine state, it is in this lagoon that we found the highest values of NO_3_^−^ and NO_2_^−^. Upstream from the lagoon, we identified two major anthropogenic pollution sources, probably linked to inefficient sanitation systems of housing (Fig. 9). In any case, these high nutrient concentrations do not seem to impact the primary producers compartment, as no particular effects on phytoplankton communities were highlighted and the lagoon is characterised by well-developed *Zostera* sp. and *Ruppia cirrhosa* meadows (Fig. 9) (Pergent-Martini et al. 1997; personal observations). Probably, the dynamics of the connection of the lagoon with the sea play an important role in diluting nutrients and suspended matter in the lagoon by enhancing water circulation and hence mitigating their effect on the biotic community (Hyman and Stephens 2020). This phenomenon was already observed in different Intermittently Closed and Opened Lagoons and Lakes (ICOLLs), where nitrogen decrease was associated with water exchanges with the adjacent marine environment (Schallenberg et al. 2010). In fact, in Balistra lagoon the sea channel outlet was constantly opened during the study period, indicating a stable connection with the sea (Fig. 8). Overall, Balistra lagoon fits well with the characteristics of an oligotrophic lagoon established in the trophic classification of aquatic ecosystems, with low Chl *a* concentration, never exceeding 3.5 µg L^−1^ (Smith et al. 1999; Smith 2003). As is typical for oligotrophic Mediterranean lagoons, in Balistra lagoon the picoplankton contribution, especially that of PE-cyan, is important and primary producers include well-developed angiosperm meadows (Bec et al. 2011). This, together with the overall dominance of diatoms in the microphytoplankton compartment and the good photosynthetic efficiency of the community, indicates a global good status of the lagoon (Smayda 1997; Pernet et al. 2012; Garrido et al. 2013b). In general, low microphytoplankton abundances were found but summer blooms of *Chaetoceros* spp. occurred. This chain-forming diatom is fundamental to sustain production and it is often dominant in other oligotrophic lagoons such as the French Ayrolle lagoon (Leruste et al. 2019). Nevertheless, some potentially harmful dinoflagellate taxa were sometimes present or even dominant, such as *Kryptoperidinium foliaceum* and *Prorocentrum micans* (Fig. 9)*.* Both these species are highly tolerant and *Kryptoperidinium* sp. in particular can bloom under very different environmental conditions, ranging from almost freshwater to hypersaline, even at the same site (Satta 2020). Despite the overall good status of the lagoon, potentially harmful species are present and their development can be boosted by nutrient pollution.

Overall, despite their comparable size, the functioning of lagoons thus proved to be different, with some shared trends nevertheless. For all three lagoons, nutrient contexts and molar ratios are consistent with typical Mediterranean lagoon conditions described by Souchu et al. (2010); DIP is consistently the most limiting factor, silicates limitation was never observed and DIN limitation sometimes occurred in summer season. We have described the current state of the three case studies lagoons, which, to date, seems to be low-impacted. However, if nothing is done, threats highlighted during the study may became cause for concern. Regarding the phytoplankton, diatoms were not correlated to the diversity index, while dinoflagellates showed an inverse relationship with Shannon’s Diversity Index, at least for Arasu and Santa Giulia lagoon. Hence, the dominance of dinoflagellates tends to homogenise the community, with high abundances or blooms of one or a few species. Since dinoflagellates and especially potentially harmful species have often been associated with anthropogenic pollution and temperature rise linked to climate change (Xiao et al. 2018; Trombetta et al. 2019), we could expect a potential degradation of these small lagoons in the future (Smayda 1997; Fischer et al. 2020; Zingone et al. 2021). Finally, based on the lagoons’ functioning descriptions provided by this study, Arasu and Santa Giulia lagoons are prone to be more susceptible to local (urbanisation) and global (climate change) changes.

### Small Lagoons in the Larger-Scale Context and Management Perspectives

Through the realisation of a first inventory and analysis of the regional coastal lagoons, we demonstrated the importance of Corsica Island as an example of coastal lagoons and especially those of small surface area. In the Mediterranean basin, only 50 lagoons have been the focus of scientific publications and many are exploited for human activities (Pérez-Ruzafa et al. 2011). However, information on small-sized lagoons at Mediterranean scale is lacking, despite the increasing demand from managers for knowledge. Small coastal lagoons not only provide numerous essential ecosystems services, but can also be considered sentinels for change, climate change or human interventions, due to their rapid response to disturbance. Yet this reactivity makes them more vulnerable to growing pressures and prone to more rapid disappearance, hence arising the need for proper management and conservation of these ecosystems (Doughty et al. 2019).

The case studies presented in this work demonstrate that local details are hugely important. This diversity is also an advantage: small lagoons constitute a mosaic of habitats, which promotes biodiversity and potentially constitutes a protection against global change by ensuring a variety of responses of these systems (Downing 2010). The local diversity, however, makes it difficult to deduce larger-scale processes for these small lagoons. Nevertheless, the great advantage is that the chosen study sites represent different conditions both in terms of hydrogeomorphology and past and current anthropogenic impact. This makes them very good examples to define similarities or dissimilarities in their functioning under different scenarios. This diversity can then reflect ecological conditions potentially present elsewhere. So, through the three different cases proposed in our study, we were able to identify general elements that are essential for the conservation and management of these small systems, in the wide range of ecological conditions that they can offer.

First, we have demonstrated that investigations on the watershed constitute a vital approach for the study of overall system dynamics of small lagoons and the interactions with adjacent systems. Surface and groundwater samples and land use analyses for the characterisation of the watershed are not systematically carried out for lagoon studies (e.g. Tuncel et al. 2007; Camacho et al. 2012). However, in order to plan an integrative management, these elements cannot be set aside and investigations on water basins and watersheds should proceed in a parallel way, especially for these poorly understood and reactive small lagoons. Fluxes, exchanges and dependence with groundwater and different markers of anthropogenic pressures should be investigated further. The watershed dynamics are fundamental to understand the functioning of lagoons and to detect potential sources of pollution linked to human exploitation in the surrounding areas (Vystavna et al. 2017; Erostate et al. 2018). Also, a focus on seasonality is important for future studies. In fact, we highlighted high tourism pressure at local scale. This is not surprising since at regional level the population in summer systematically doubles (at least) the resident population since decades: for instance, in 2021 the Island hosted 371,000 tourists, over a population of 346,610 residents (DREAL Corse; ^©^Insee, https://www.insee.fr). This suggests that during summer the lagoons are probably subjected to higher nutrient inputs and hydric stress, due to increased water demand for usages (Orsoni 2001; Collos et al. 2003). Hence, this justifies the need of attention towards seasonal dynamics for future research.

Secondly, connection with the marine environment is also a crucial point in the small lagoons’ functioning and it is essential to take it into account for effective management (Schallenberg et al. 2010; Biggs et al. 2015; Jones et al. 2018). With the progression of climate change, since rainfall events are expected to become less frequent and more intense, these reactive small shallow lagoons may then increasingly experience confinement or isolation. In turn, this can lead to frequent episodes of drought due to evaporation and have cascade effects at different trophic levels, such as changes in the phytoplankton community and the emergence of potentially harmful stress-tolerant taxa, loss of angiosperms and fish mortality (Angeler et al. 2010; Rojo et al. 2012).

Thirdly, our study focused on a few biotic and abiotic parameters and especially phytoplankton communities for the biotic component. Despite phytoplankton being an excellent indicator of the ecosystem’s status, we suggest that an ecosystem-based approach should be taken into account for future studies, especially considering lagoons’ connection to adjacent systems (Ligorini et al. 2022b). This ecosystem-based approach is becoming more and more widely applied, especially in unstable transitional ecosystems, and it would enable a better understanding of ecosystem functioning at multiple levels and help with management decisions (Walker et al. 2006; Hobbs et al. 2009; Biggs et al. 2015; García-Ayllón, 2017; De Wit et al. 2020). Figure 9 offers a preface to this approach, which is essential to answer managers' question regarding the status and functioning of these poorly known small lagoons.

Additionally, these small systems should be considered as socio-ecosystems; one of the first stages to consider when planning management strategies is the assessment of ecosystem services provided by the ecosystem and their prioritisation, combining scientific and technical expertise with stakeholders’ perceptions and demands in order to help with political decisions (De Wit et al. 2020). Ecological issues cannot be completely independent of social-economic ones: the prior establishment of a “desired state” can allow the centralisation of decisional power and the allocation of financial resources in an adapted management roadmap (De Wit et al. 2020). For example, Arasu and Santa Giulia lagoons are set in a high value context in terms of tourism, some business activities have developed on their borders, and they are thus subjected to a political demand for the preservation of these activities, which should therefore be taken into account in management decisions. An important issue then arises: is the good ecological status of lagoons an added value for seaside tourism activities? If so, it is of fundamental importance to create platforms or any kind of exchange tool or space in order to share the scientific knowledge acquired with the multiple actors involved, such as managers and stakeholders, and hence to encourage discussion and consensus towards a commonly established desired state (Maciejewski et al. 2016; De Wit et al. 2020, 2021; Pérez-Ruzafa et al. 2020).

Moreover, small lagoons are not included in the WFD, so no regular scientific surveys are carried out in this context and no “good status” goal has been established, meaning the acquisition of scientific knowledge is difficult. Unprotected sites are more vulnerable to climate change and other threats (Leberger et al. 2020). All three lagoons in this study are subject to protection status (Natura 2000 sites), but the deployment of actual management plans and actions is still in its initial phase. Despite the accumulation of protection statuses and labels, the application of efficient management can be problematic due to complex property regimes (De Wit et al. 2021; OEC 2022). The fragmentation of properties and their private status imply dispersed and disconnected management that can therefore prove difficult to implement or ineffective, as is the case notably for Balistra lagoon (Pérez-Ruzafa et al. 2020; De Wit et al. 2021). Meanwhile, efforts already undertaken by some public institutions, such as the CdL, to acquire Corsican lagoons should be carried on to centralise ownership and management and guarantee the prevention of habitat destruction by the uncontrolled progression of urban and tourism development (De Wit et al. 2021). Arasu lagoon is under mixed ownership by multiple private and public owners. This entails difficulties in terms of management because it requires the coordination and mutual agreement of the numerous actors involved and the satisfaction of different political and ecological demands, making it difficult to prioritise the expertise of managers. In Santa Giulia lagoon, despite its centralised ownership, there are some difficulties, mostly linked to unauthorised management actions carried out independently by stakeholders. These actions are carried out outside the environmental management legal framework and have caused various problems, including the reduction of the surface area of the water basin.

Finally, given all these premises, there is a need for the development of appropriate tools to provide guidance for the management of these small systems. As we highlighted, unauthorised interventions should be prevented, since they can disrupt effective scientific and management programmes by altering the natural behaviour of the system, as it was the case for the small Koggala lagoon (Sri Lanka) (Gunaratne et al. 2011). Nevertheless, human intervention is not to be demonised and is often central for restoration purposes. For example, there is evidence that implementation of active restoration actions and artificial infrastructures can be used to control lagoon functioning, reduce confinement and rehabilitate hydrogeomorphological features in small lagoons (Gunaratne et al. 2011; Camacho et al. 2012). Deeply altered small lagoon, similar to Arasu lagoon, could be potential candidates for active restoration. To consider such interventions, management of sea channel openings or other actions, multi-factorial modelling can be a helpful tool to design potential scenarios and the reactions of ecosystems (e.g. GAMELag, Pete et al. 2020). Our work is hence a valuable contribution for the potential application of this kind of modelling on small-surface lagoons, since this requires a deep knowledge base of the studied systems and of their responses towards local and global change (Gunaratne et al. 2011; Jones et al. 2018; Doughty et al. 2019; Pérez-Ruzafa et al. 2019; Teixeira and Solari 2020). These models are an important tool to support discussions between the actors involved and hence achieve consensus on appropriate and well thought-out management plans and decisions.

A major challenge lies ahead for these small-sized lagoons: their importance is acknowledged, but proper management is still lacking and raises many questions. This study is an essential first step towards the understanding of the ecological functioning of these small-sized lagoon ecosystems (Fig. 9). It has provided the first inventory of Corsican lagoons and has enabled us to point out the importance of these systems at regional scale and in the Mediterranean area as a whole. Through the investigation of three case studies, it has provided useful elements for applications elsewhere at larger scale. This work demonstrates how complex, diverse and reactive these small lagoons are, making them not only perfect sentinels of global change, but also highly vulnerable. We have highlighted the need for promoting their conservation through the acquisition of fundamental knowledge in a connected and dynamic context that will constitute a good tool for offering guidance for management, especially within a challenging perspective of climate change and increasing anthropogenic threats.

## Supplementary Information

Below is the link to the electronic supplementary material.

Supplementary file1 (DOCX 526 KB)

## Data Availability

The datasets generated and/or analysed during the current study are available from the corresponding author on reasonable request.
