# Supplementary material for: Small Mediterranean coastal Lagoons Under Threat: Hydro-ecological Disturbances and Local Anthropogenic Pressures (Size Matters)
Source: Estuaries Coast. 2023 Feb 27:1–24. Online ahead of print. doi: 10.1007/s12237-023-01182-1 (PMC9970120; doi:10.1007/s12237-023-01182-1)
Supplement: Supplementary file 1 — Supplementary file1 (DOCX 526 KB) [file 12237_2023_1182_MOESM1_ESM.docx]

**Estuaries and Coasts**

**Small Mediterranean coastal Lagoons Under Threat: Hydro-Ecological Disturbances and Local Anthropogenic Pressures (Size Matters)**

**Viviana Ligorini ^1,2,3*^, Eléa Crayol ^1,3^, Frédéric Huneau ^1,3^, Emilie Garel ^1,3^, Nathalie Malet ^4^, Marie Garrido ^5^, Louise Simon^1,2,3^, Philippe Cecchi ^6^, Vanina Pasqualini ^1,2,3^**

*^a^ Université de Corse Pascal Paoli, Campus Grimaldi, BP52, 20250 Corte, France*

*^b^ CNRS, UAR 3514 Stella Mare, Cordon Lagunaire de la Marana, lieu-dit U Casone, 20620 Biguglia, France*

*^c^ CNRS, UMR 6134 SPE, BP 52, 20250 Corte, France*

*^d^ Ifremer, Laboratoire Environnement Ressources Provence-Azur-Corse (LER/PAC), Implantation de Bastia, Z.I. Furiani, Immeuble Agostini, 20600 Bastia, France*

*^e^ Environmental Agency of Corsica, 14 Avenue Jean Nicoli, 20250 Corte, France*

*^g^ MARBEC, Univ. Montpellier, CNRS, Ifremer, IRD, Montpellier, France*

^*^ Corresponding author

*E-mail address:* [ligorini_v@univ-corse.fr](mailto:ligorini_v@univ-corse.fr)

<https://orcid.org/0000-0001-9846-568X>

Submission date: 20 January 2023

Electronic Supplementary Material.

**Table S1.** Floristic list, by year and season, of all phytoplankton taxonomic units identified in each lagoon during microscopy analyses. The presence of a taxonomic unit is identified by an “X” in the corresponding case, while empty cases indicate the absence of the taxonomic entity. “Diat.”, “Dino.” and “Und.” stand for “Diatom”, “Dinoflagellate” and “Undetermined”, respectively.

|  |  | **Arasu** | | | | | | | | **Santa Giulia** | | | | | | | | **Balistra** | | | | | | | |
| --- | --- | --- | --- | --- | --- | --- | --- | --- | --- | --- | --- | --- | --- | --- | --- | --- | --- | --- | --- | --- | --- | --- | --- | --- | --- |
|  |  | **2020** | | | | **2021** | | | | **2020** | | | | **2021** | | | | **2020** | | | | **2021** | | | |
|  |  | **Winter** | **Spring** | **Summer** | **Autumn** | **Winter** | **Spring** | **Summer** | **Autumn** | **Winter** | **Spring** | **Summer** | **Autumn** | **Winter** | **Spring** | **Summer** | **Autumn** | **Winter** | **Spring** | **Summer** | **Autumn** | **Winter** | **Spring** | **Summer** | **Autumn** |
| *Chaetoceros* sp. 1 |  | **X** | **X** |  |  |  |  |  |  |  |  |  |  |  |  |  |  |  |  |  |  |  |  |  |  |
| *Chaetoceros* sp. 2 |  |  | **X** | **X** | **X** |  |  | **X** |  |  | **X** |  |  |  |  | **X** | **X** |  | **X** | **X** |  |  | **X** | **X** | **X** |
| *Cheatoceros peruvianus* |  |  |  |  |  |  |  |  |  |  |  |  |  |  |  |  |  |  |  |  | **X** |  |  |  |  |
| *Achnanthes* sp. |  | **X** |  |  |  |  |  |  |  | **X** | **X** |  | **X** | **X** | **X** | **X** |  |  | **X** |  |  |  |  |  |  |
| *Cylindrotheca closterium* |  | **X** | **X** | **X** | **X** |  |  |  |  |  |  |  | **X** |  |  |  |  |  | **X** | **X** | **X** |  |  |  |  |
| *Amphora* sp. |  | **X** | **X** | **X** | **X** | **X** | **X** | **X** | **X** | **X** | **X** |  | **X** | **X** | **X** | **X** | **X** | **X** | **X** | **X** | **X** | **X** | **X** | **X** | **X** |
| *Cocconeis* sp. |  | **X** |  |  |  |  | **X** |  | **X** | **X** |  |  |  | **X** | **X** | **X** | **X** | **X** | **X** | **X** | **X** | **X** | **X** | **X** | **X** |
| *Navicula* spp. |  | **X** | **X** | **X** | **X** | **X** | **X** | **X** | **X** | **X** | **X** |  | **X** | **X** | **X** | **X** | **X** | **X** | **X** | **X** | **X** | **X** | **X** | **X** | **X** |
| *Diploneis* sp. |  | **X** | **X** | **X** | **X** | **X** | **X** | **X** |  |  |  |  |  | **X** |  |  |  | **X** | **X** | **X** | **X** | **X** | **X** | **X** |  |
| *Gyrosigma/Pleurosigma* sp. |  | **X** | **X** |  | **X** |  |  | **X** | **X** | **X** | **X** |  | **X** | **X** | **X** |  |  | **X** | **X** | **X** | **X** | **X** | **X** | **X** | **X** |
| *Petroneis* sp. |  | **X** |  |  |  |  | **X** | **X** |  | **X** |  |  | **X** | **X** | **X** | **X** |  |  |  |  |  |  | **X** | **X** |  |
| *Surirella* sp. |  | **X** |  |  | **X** | **X** |  | **X** |  | **X** | **X** |  | **X** | **X** | **X** | **X** | **X** | **X** | **X** |  | **X** |  | **X** | **X** |  |
| *Entomoneis* sp. |  | **X** |  |  |  | **X** | **X** | **X** | **X** | **X** | **X** |  | **X** | **X** | **X** |  |  | **X** | **X** | **X** | **X** |  | **X** | **X** | **X** |
| *Fragilaria/Synedra* sp. 1 |  | **X** | **X** | **X** | **X** | **X** | **X** |  |  | **X** | **X** |  | **X** | **X** |  |  |  | **X** | **X** | **X** | **X** | **X** |  |  |  |
| *Fragilaria/Synedra* sp. 2 |  |  | **X** |  |  |  | **X** | **X** | **X** |  |  |  |  | **X** | **X** | **X** | **X** |  |  |  |  |  | **X** | **X** | **X** |
| Small centric diatoms |  |  | **X** |  |  | **X** | **X** | **X** | **X** |  | **X** |  |  |  |  |  | **X** |  | **X** |  |  |  | **X** | **X** | **X** |
| Small pennate diatoms |  |  | **X** | **X** |  |  |  |  |  |  | **X** |  |  |  |  | **X** |  |  | **X** | **X** |  |  |  |  |  |
| *Coscinodiscus granii* |  |  |  | **X** |  |  |  |  |  |  |  |  |  |  |  |  |  |  |  |  |  |  |  |  |  |
| *Coscinodiscus* sp. |  |  |  |  |  | **X** |  | **X** |  |  |  |  |  |  |  |  |  |  |  |  | **X** |  |  |  |  |
| *Nitzschia* sp. |  |  |  | **X** |  |  |  | **X** | **X** |  | **X** |  | **X** | **X** | **X** | **X** | **X** |  | **X** |  |  |  | **X** | **X** | **X** |
| *Nitzschia sigmoidea* |  |  |  |  |  |  |  |  |  |  |  |  |  |  |  |  |  | **X** |  |  |  |  |  |  |  |
| *Nitzschia longissima* |  |  |  |  |  |  |  |  |  |  |  |  |  |  |  |  |  | **X** |  |  |  | **X** |  |  |  |
| *Nitzchia scalaris* |  |  |  |  |  |  |  |  |  |  |  |  |  |  |  |  |  |  |  |  |  | **X** |  |  |  |
| *Rhopalodia/Campylodiscu*s sp. |  |  |  |  |  |  |  |  |  | **X** |  |  |  |  |  |  |  |  |  |  |  |  |  |  |  |
| *Melosira* sp. |  |  |  |  |  | **X** |  |  |  | **X** | **X** |  |  | **X** | **X** |  |  |  | **X** |  | **X** |  |  | **X** |  |
| *Lyrella* sp. |  |  |  |  |  |  |  |  |  |  |  |  | **X** |  | **X** |  |  |  |  | **X** | **X** | **X** | **X** | **X** |  |
| *Licmophora* sp. |  |  |  |  |  |  |  | **X** | **X** |  |  |  | **X** | **X** |  | **X** | **X** |  |  |  | **X** |  | **X** | **X** | **X** |
| *Hantzchia* sp. |  |  |  |  |  |  |  |  |  |  |  |  |  | **X** |  |  |  |  |  |  |  | **X** |  |  |  |
| *Pinnularia* sp. |  |  |  |  |  |  |  |  |  |  |  |  |  | **X** |  |  |  |  |  |  |  |  |  |  |  |
| *Thalassionema* sp. |  |  |  |  |  |  |  |  |  |  |  |  |  |  |  |  |  |  | **X** |  |  |  |  |  |  |
| *Grammatophora* sp. |  |  |  |  |  |  |  |  |  |  |  |  |  |  |  |  |  |  | **X** |  |  |  |  |  |  |
| *Striatella unipunctata* |  |  |  |  |  |  |  |  |  |  |  |  |  |  |  |  |  |  |  | **X** |  |  |  |  |  |
| *Striatella* sp. 1 |  |  |  |  |  |  | **X** | **X** |  |  |  |  |  |  |  |  |  |  |  |  |  |  |  |  |  |
| *Striatella* sp. 2 |  |  |  |  |  |  |  | **X** |  |  |  |  |  |  |  |  |  |  |  |  |  |  |  |  | **X** |
| *Leptocylindrus* sp. |  |  |  |  |  |  |  | **X** |  |  |  |  |  |  |  |  |  |  |  |  |  |  | **X** |  |  |
| *Cerataulina pelagica* |  |  |  |  |  |  |  |  |  |  |  |  |  |  |  |  |  |  |  |  |  |  | **X** |  |  |
| *Bacillaria* sp. |  |  |  |  |  |  |  |  |  |  |  |  |  |  |  |  |  |  |  |  |  |  |  |  | **X** |
| Diat. Und. 1 |  |  | **X** |  |  |  |  |  |  |  |  |  |  |  |  |  |  |  |  |  |  |  |  |  |  |
| Diat. Und. 2 |  | **X** |  |  |  |  |  |  |  |  |  |  |  |  |  |  |  |  |  |  |  |  |  |  |  |
| Diat. Und. 3 |  | **X** |  |  |  |  |  |  |  |  |  |  |  |  |  |  |  |  |  |  |  |  |  |  |  |
| Diat. Und. 4 |  | **X** |  |  |  |  |  |  |  |  |  |  |  |  |  |  |  |  |  |  |  |  |  |  |  |
| Diat. Und. 5 |  | **X** |  |  |  |  |  |  |  |  |  |  |  |  |  |  |  |  |  |  |  |  |  |  |  |
| Diat.Und.6 |  |  |  |  |  |  |  |  |  |  |  |  |  |  |  |  |  | **X** |  |  |  |  |  |  |  |
| Diat.Und.7 |  |  |  |  |  |  |  |  |  |  |  |  |  |  |  |  |  |  |  | **X** |  |  |  |  |  |
| Diat.Und.8 |  |  |  |  |  |  |  | **X** |  |  |  |  |  |  |  |  |  |  |  |  |  |  |  |  |  |
| Diat.Und.9 |  |  | **X** |  |  |  |  |  |  | **X** |  |  |  |  |  |  |  |  |  |  |  |  |  |  |  |
| Diat.Und.10 |  |  |  |  |  |  |  |  | **X** |  |  |  |  |  |  |  |  |  |  |  |  |  |  |  |  |
| Diat.Und.11 |  |  |  |  |  |  |  |  |  |  |  |  |  |  |  |  |  |  | **X** |  |  |  |  |  |  |
| *Prorocentrum micans* |  | **X** | **X** | **X** | **X** | **X** | **X** | **X** | **X** | **X** | **X** |  |  | **X** | **X** | **X** | **X** |  | **X** | **X** | **X** |  | **X** | **X** | **X** |
| *Prorocentrum cordatum* |  | **X** | **X** | **X** | **X** |  | **X** |  | **X** |  | **X** |  | **X** |  |  |  |  | **X** | **X** |  |  |  |  |  |  |
| *Prorocentrum lima* |  |  |  | **X** |  | **X** | **X** | **X** |  |  |  |  |  |  |  |  |  |  |  |  |  |  |  |  |  |
| *Prorocentrum* sp. |  |  |  |  |  |  |  |  |  |  |  |  |  | **X** |  |  |  |  |  |  |  |  |  |  |  |
| *Gymnodinium* sp. |  | **X** |  |  |  |  |  |  |  |  |  |  | **X** |  |  |  |  |  | **X** |  |  |  |  |  |  |
| Gymnodiniales |  |  |  |  |  |  | **X** |  |  |  |  |  |  |  |  |  |  |  |  |  |  |  | **X** |  |  |
| *Gymnodinium/Kaarenia/Kryptoperidinium* sp. |  |  |  |  |  |  |  |  |  |  |  |  |  |  |  |  |  | **X** |  |  |  |  |  |  |  |
| *Kryptoperidinium foliaceum* |  |  |  |  |  | **X** | **X** | **X** | **X** |  |  |  |  | **X** |  |  | **X** |  | **X** |  | **X** | **X** | **X** | **X** |  |
| *Dinophysis* sp. |  | **X** |  | **X** | **X** |  |  |  |  |  |  |  |  |  | **X** |  |  | **X** | **X** | **X** |  |  |  |  |  |
| *Dinophysis caudata* |  |  |  |  |  |  |  |  |  |  |  |  |  |  |  |  |  |  | **X** | **X** | **X** |  |  |  |  |
| *Diplopsalis* sp. |  | **X** | **X** |  | **X** |  |  |  | **X** | **X** |  |  |  |  |  | **X** | **X** | **X** |  |  |  |  |  | **X** | **X** |
| *Gonyaulax* sp. |  | **X** |  |  | **X** |  |  | **X** | **X** |  |  |  |  |  |  |  |  |  | **X** |  |  |  | **X** | **X** | **X** |
| *Gonyaulax spinifera* |  |  |  |  |  |  |  |  |  | **X** |  |  |  |  |  |  |  |  |  |  |  |  |  |  |  |
| *Protoperidiunium* sp. 1 |  |  | **X** |  |  |  |  |  |  |  |  |  |  |  |  |  |  |  | **X** | **X** |  |  |  |  |  |
| *Protoperidinium* sp. 2 |  |  | **X** | **X** | **X** |  |  | **X** |  |  |  |  |  |  |  |  |  |  |  | **X** | **X** |  |  | **X** | **X** |
| *Protoperidinium bipes* |  |  |  |  |  |  |  |  | **X** |  |  |  |  |  |  |  |  |  |  |  |  |  |  |  |  |
| *Protoperidinium brevipes* |  |  |  |  |  | **X** | **X** | **X** | **X** |  |  |  | **X** | **X** |  |  |  |  |  |  |  |  | **X** | **X** |  |
| *Protoperidinium crassipes* |  |  |  |  |  |  | **X** |  |  |  |  |  |  |  |  |  |  |  |  |  |  |  |  |  |  |
| *Protoperidinium divergens* |  |  |  |  |  |  |  |  |  |  |  |  |  |  |  |  |  |  |  |  |  |  | **X** | **X** |  |
| *Akashiwo sanguinea* |  |  |  | **X** | **X** | **X** | **X** | **X** | **X** |  |  |  |  |  |  | **X** | **X** |  |  | **X** |  |  |  | **X** | **X** |
| *Scrippsiella* sp. |  |  |  |  |  |  |  |  | **X** |  |  |  | **X** |  |  |  |  |  |  |  | **X** |  |  | **X** | **X** |
| *Scrippsiella trocoïdea* |  |  |  |  |  |  |  |  |  |  |  |  |  |  |  |  |  |  | **X** |  |  |  |  |  |  |
| *Ceratium furca* |  |  |  |  |  |  |  |  |  |  |  |  |  |  |  |  |  |  |  | **X** | **X** |  |  | **X** |  |
| *Ceratium fusus* |  |  |  |  |  |  |  |  |  |  |  |  |  |  |  |  |  |  |  |  | **X** |  |  |  |  |
| *Alexandrium* sp. |  |  |  |  |  |  | **X** | **X** | **X** |  |  |  |  | **X** | **X** |  | **X** |  |  |  |  |  | **X** | **X** |  |
| *Gyrodinium* sp. |  |  |  |  |  |  | **X** |  |  |  |  |  |  |  |  |  |  |  |  |  |  |  |  |  |  |
| Dino. Und. 1 |  | **X** |  |  |  |  |  |  |  |  |  |  |  |  |  |  |  |  |  |  |  |  |  |  |  |
| Dino. Und. 2 |  |  | **X** |  |  |  |  |  |  |  |  |  |  |  |  |  |  |  |  |  |  |  |  |  |  |
| Dino. Und. 3 |  |  |  | **X** |  |  |  |  |  |  |  |  |  |  |  |  |  |  |  |  |  |  |  |  |  |
| Dino. Und. 4 |  |  |  | **X** |  |  |  |  |  |  |  |  |  |  |  |  |  |  |  |  |  |  |  |  |  |
| Dino. Und. 5 |  |  |  |  | **X** |  |  |  |  |  |  |  |  |  |  |  |  |  |  |  |  |  |  |  |  |
| Dino. Und. 6 |  |  | **X** | **X** | **X** | **X** | **X** | **X** | **X** |  | **X** |  | **X** | **X** | **X** |  | **X** |  | **X** | **X** | **X** | **X** | **X** |  | **X** |
| Dino. Und. 7 |  |  |  |  |  |  |  |  |  |  |  |  |  |  | **X** |  |  |  |  |  |  |  |  |  |  |
| Dino. Und. 8 |  |  |  |  |  |  |  |  |  |  |  |  |  |  |  |  |  | **X** |  |  |  |  |  |  |  |
| Dino. Und. 9 |  |  |  |  |  |  |  |  |  |  |  |  |  |  |  |  |  | **X** |  |  |  |  |  |  |  |
| Dino. Und. 10 |  |  |  |  |  |  |  |  |  |  |  |  |  |  |  |  |  |  |  | **X** |  |  |  |  |  |
| Cyanophyceae |  | **X** |  | **X** |  |  | **X** | **X** |  | **X** |  |  | **X** | **X** |  | **X** | **X** | **X** | **X** |  |  |  | **X** |  | **X** |
| *Merismopedia* sp. |  |  | **X** |  |  |  |  |  |  |  |  |  |  |  |  |  |  |  |  |  |  |  |  |  |  |
| *Lyngbya* sp. |  |  |  |  |  |  |  |  | **X** |  | **X** |  | **X** |  |  |  |  |  |  |  |  | **X** |  |  |  |
| *Anabaena* sp. |  |  |  |  |  |  | **X** |  |  |  |  |  |  |  |  |  |  |  |  |  |  |  |  |  |  |
| *Oscillatoria* sp. |  |  |  |  |  |  |  |  |  |  |  |  |  | **X** |  |  |  |  |  |  |  |  |  |  |  |
| Chlorophyceae |  | **X** |  |  |  |  |  |  |  | **X** |  |  |  |  |  |  |  | **X** |  |  |  |  |  |  |  |
| Prasinophyceae |  | **X** |  |  |  |  |  |  |  |  |  |  |  |  |  |  |  |  |  | **X** |  |  |  |  |  |
| Euglenophyceae |  | **X** | **X** | **X** |  |  |  | **X** |  |  |  |  |  |  |  |  | **X** | **X** | **X** | **X** |  |  | **X** |  |  |
| Chrysophyceae Und. 1 |  | **X** |  |  |  |  |  |  |  |  |  |  |  |  |  |  |  |  | **X** | **X** |  |  |  |  |  |
| *Synura* sp. |  |  |  | **X** | **X** | **X** | **X** | **X** | **X** |  |  |  | **X** |  |  |  |  |  |  |  |  |  |  |  |  |
| *Mallomonas* sp. |  |  |  |  |  | **X** |  |  |  |  |  |  |  |  |  |  |  |  |  |  |  |  |  |  |  |
| Cryptophyceae |  |  | **X** | **X** |  | **X** |  | **X** | **X** |  | **X** |  | **X** |  | **X** |  | **X** |  |  |  |  | **X** |  |  | **X** |
| *Rhadosphaera* sp. |  |  |  |  |  |  |  |  |  |  |  |  |  |  |  |  |  |  |  |  | **X** |  |  |  |  |
| *Apedinella radians* |  |  |  |  |  |  |  | **X** |  |  |  |  |  | **X** |  |  |  |  |  |  |  |  |  |  |  |
| Und.1 |  | **X** |  |  |  |  |  |  |  |  |  |  |  |  |  |  |  |  |  |  |  |  |  |  |  |
| Und.2 |  | **X** |  |  |  |  |  |  |  |  |  |  |  |  |  |  |  |  |  |  |  |  |  |  |  |
| Und.3 |  | **X** |  |  |  |  |  |  |  |  |  |  |  |  |  |  |  |  |  |  |  |  |  |  |  |
| Und.4 |  |  |  | **X** |  |  |  |  |  |  |  |  |  |  |  |  |  |  |  |  |  |  |  |  |  |
| Und.5 |  |  |  | **X** |  |  |  |  |  |  |  |  |  |  |  |  |  |  |  |  |  |  |  |  |  |
| Und.6 |  |  |  |  | **X** |  |  |  |  |  |  |  |  |  |  |  |  |  |  |  |  |  |  |  |  |
| Und. 7 |  |  |  | **X** |  |  |  |  |  |  |  |  |  |  |  |  |  |  |  | **X** |  |  |  |  |  |
| Und. 8 |  |  |  |  | **X** |  |  |  |  |  |  |  |  |  |  |  |  |  |  |  |  |  |  |  |  |
| Und. 9 |  |  |  |  |  |  |  | **X** |  |  |  |  |  |  |  |  |  |  |  |  |  |  |  |  |  |
| Und. 10 |  |  |  |  |  |  | **X** |  |  |  |  |  |  |  |  |  |  |  |  |  |  |  |  |  |  |
| Und. 11 |  |  |  |  |  |  |  |  |  | **X** |  |  |  |  |  |  |  |  |  |  |  |  |  |  |  |
| Und. 12 |  |  |  |  |  |  |  |  |  | **X** |  |  |  |  |  |  |  |  |  |  |  |  |  |  |  |
| Und. 13 |  |  |  |  |  |  |  |  |  |  |  |  | **X** |  |  |  |  |  |  |  |  |  |  |  |  |
| Und. 14 |  |  |  |  |  |  |  |  |  |  |  |  |  |  |  |  |  |  | **X** |  |  |  |  |  |  |


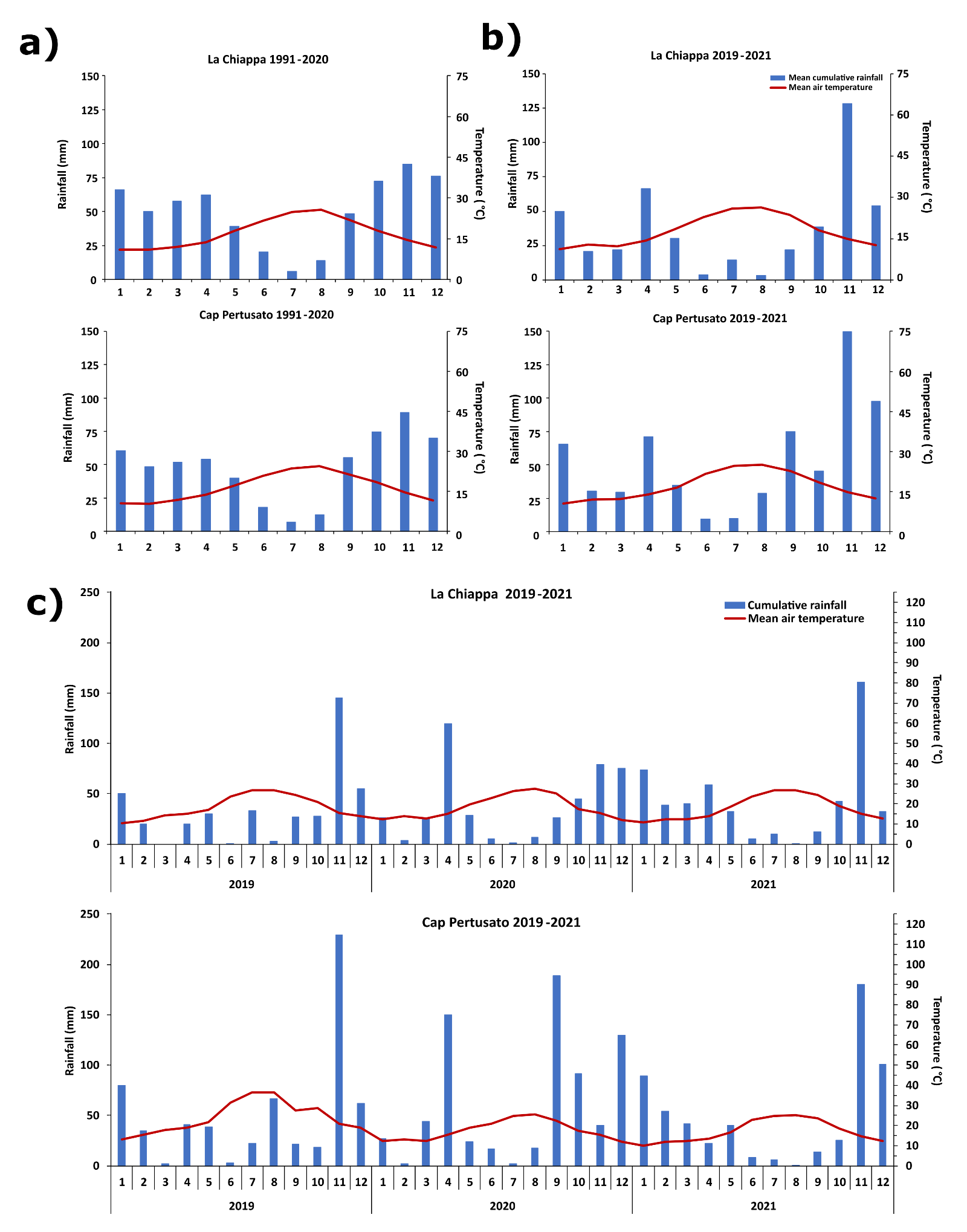


**Fig. S1** Meteorological context for the two weather stations (La Chiappa and Cap Pertusato). Left scale corresponds to rainfall (mm, blue bars) and right scale to temperature (°C, red line) for the ombrothermic diagrams over **a** the reference period (1991-2020) and **b** the study period (2019-2021), and **c** the representation of monthly mean values for the two sampling years (2020-2021) and antecedent year (2019).

**Monthly analysis of meteorological context for the study period:**

At monthly scale, for both stations mean air temperatures followed the seasonal pattern. November 2019 was particularly rainy (cumulative rainfall: 146 mm and 229 mm for La Chiappa and Cap Pertusato stations respectively; Supplementary, Fig. S1c). 2020’s winter was especially dry and warm, with cumulative rainfall reaching only 3.4 mm and 2.2 mm and mean air temperature of 14°C and 13°C for La Chiappa and Cap Pertusato stations respectively (Supplementary, Fig. S1c). In 2020, spring rainfall was concentrated in April, which was the rainiest spring month of the year for both stations (cumulative rainfall: 120 mm (La Chiappa) and 150 mm (Cap Pertusato); Supplementary, Fig. S1c). 2021 showed a different pattern than 2020, mainly with more humid and cool winter (February 2021 cumulative rainfall and mean air temperature: 39 mm and 12°C (La Chiappa), 55 mm and 12°C (Cap Pertusato); Supplementary, Fig. S1c), a quite dry spring, but with longer rainy period than 2020 and autumn rainfall concentrated in November (161 mm (La Chiappa) and 180 mm (Cap Pertusato); Supplementary, Fig. S1c). Such a variability is typical of Mediterranean climate.


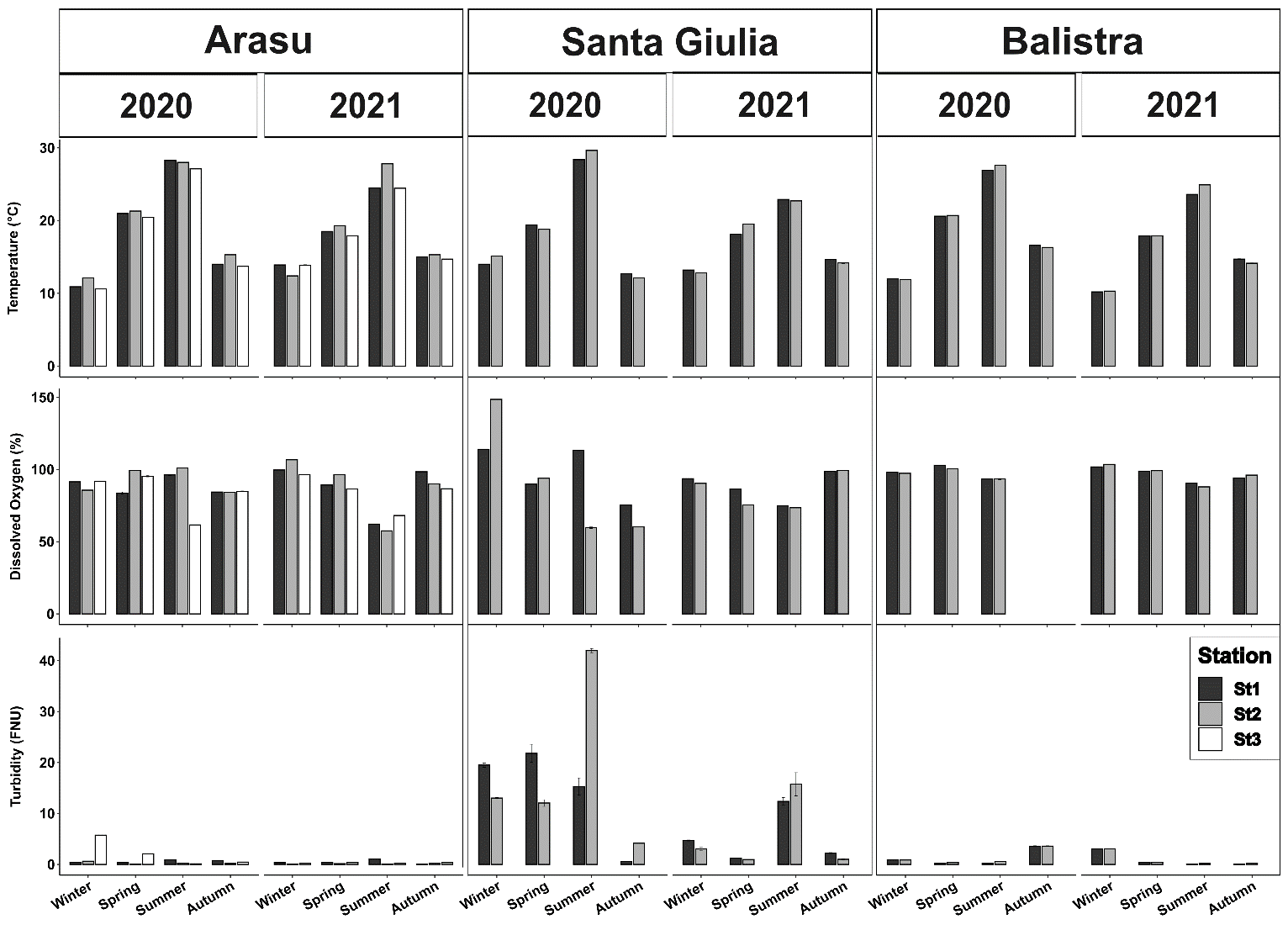


**Fig. S2** Temperature (°C), dissolved oxygen concertation (% saturation) and turbidity (FNU) seasonal values for the three studied lagoons in 2020 and 2021, with error bars representing Standard Error over the three replicates measured *in situ.*
